# Gap-free genome assembly and *CYP450* gene family analysis reveal the biosynthesis of anthocyanins in *Scutellaria baicalensis*

**DOI:** 10.1093/hr/uhad235

**Published:** 2023-11-17

**Authors:** Tianlin Pei, Sanming Zhu, Weizhi Liao, Yumin Fang, Jie Liu, Yu Kong, Mengxiao Yan, Mengying Cui, Qing Zhao

**Affiliations:** Shanghai Key Laboratory of Plant Functional Genomics and Resources, CAS Center for Excellence in Molecular Plant Sciences Chenshan Plant Science Research Center, Shanghai Chenshan Botanical Garden, Shanghai, 201602, China; State Key Laboratory of Plant Molecular Genetics, CAS Center for Excellence in Molecular Plant Sciences, Chinese Academy of Sciences, Shanghai, 200032, China; Shanghai Key Laboratory of Plant Functional Genomics and Resources, CAS Center for Excellence in Molecular Plant Sciences Chenshan Plant Science Research Center, Shanghai Chenshan Botanical Garden, Shanghai, 201602, China; National Key Laboratory of Wheat Improvement, College of Life Sciences, Shandong Agricultural University, Taian, 271018, China; Shanghai Key Laboratory of Plant Functional Genomics and Resources, CAS Center for Excellence in Molecular Plant Sciences Chenshan Plant Science Research Center, Shanghai Chenshan Botanical Garden, Shanghai, 201602, China; Shanghai Key Laboratory of Plant Molecular Sciences, College of Life Sciences, Shanghai Normal University, Shanghai, 200234, China; Shanghai Key Laboratory of Plant Functional Genomics and Resources, CAS Center for Excellence in Molecular Plant Sciences Chenshan Plant Science Research Center, Shanghai Chenshan Botanical Garden, Shanghai, 201602, China; Shanghai Key Laboratory of Plant Functional Genomics and Resources, CAS Center for Excellence in Molecular Plant Sciences Chenshan Plant Science Research Center, Shanghai Chenshan Botanical Garden, Shanghai, 201602, China; Shanghai Key Laboratory of Plant Functional Genomics and Resources, CAS Center for Excellence in Molecular Plant Sciences Chenshan Plant Science Research Center, Shanghai Chenshan Botanical Garden, Shanghai, 201602, China; Shanghai Key Laboratory of Plant Functional Genomics and Resources, CAS Center for Excellence in Molecular Plant Sciences Chenshan Plant Science Research Center, Shanghai Chenshan Botanical Garden, Shanghai, 201602, China; Shanghai Key Laboratory of Plant Functional Genomics and Resources, CAS Center for Excellence in Molecular Plant Sciences Chenshan Plant Science Research Center, Shanghai Chenshan Botanical Garden, Shanghai, 201602, China; Shanghai Key Laboratory of Plant Functional Genomics and Resources, CAS Center for Excellence in Molecular Plant Sciences Chenshan Plant Science Research Center, Shanghai Chenshan Botanical Garden, Shanghai, 201602, China; State Key Laboratory of Plant Molecular Genetics, CAS Center for Excellence in Molecular Plant Sciences, Chinese Academy of Sciences, Shanghai, 200032, China

## Abstract

*Scutellaria baicalensis* Georgi, a member of the Lamiaceae family, is a widely utilized medicinal plant. The flavones extracted from *S. baicalensis* contribute to numerous health benefits, including anti-inflammatory, antiviral, and anti-tumor activities. However, the incomplete genome assembly hinders biological studies on *S. baicalensis*. This study presents the first telomere-to-telomere (T2T) gap-free genome assembly of *S. baicalensis* through the integration of Pacbio HiFi, Nanopore ultra-long and Hi-C technologies. A total of 384.59 Mb of genome size with a contig N50 of 42.44 Mb was obtained, and all sequences were anchored into nine pseudochromosomes without any gap or mismatch. In addition, we analysed the major cyanidin- and delphinidin-based anthocyanins involved in the determination of blue-purple flower using a widely-targeted metabolome approach. Based on the genome-wide identification of *Cytochrome P450 (CYP450)* gene family, three genes (*SbFBH1*, *2*, and *5*) encoding flavonoid 3′-hydroxylases (F3′Hs) and one gene (*SbFBH7*) encoding flavonoid 3′5′-hydroxylase (F3′5′H) were found to hydroxylate the B-ring of flavonoids. Our studies enrich the genomic information available for the Lamiaceae family and provide a toolkit for discovering *CYP450* genes involved in the flavonoid decoration.

## Introduction

*Scutellaria baicalensis* Georgi, commonly referred to as Huang Qin in Chinese and also known as Chinese skullcap, is a perennial herb indigenous to regions encompassing China, Mongolia, Japan, the Russian Federation, and the Korean peninsula [1]. This plant belongs to the Lamiaceae family and has a long history of utilization in traditional Chinese medicine for various therapeutic purposes like diarrhea, dysentery, hypertension, and inflammation [2]. Modern pharmacological investigations have confirmed the potential neuro- and hepato-protective, as well as antimicrobial, anti-inflammatory, antioxidative, antiviral, and antitumor activities [3].

*S. baicalensis* is rich in flavonoids which contribute most health benefits, including baicalein, wogonin, baicalin, and wogonoside [4]. Lacking a 4′-OH group on their B-rings differentiates these flavones from more classical 4′-hydroxyflavones like scutellarein and scutellarin, which are widely found in *S. baicalensis* aerial tissues [5]. In recent years, many studies have been dedicated to exploring the biochemical roles and evolutionary trajectories of genes pivotal to the synthesis of baicalein and wogonin as well as their modifications (*O*-glycosylation and *O*-methylation) in *S. baicalensis*[6–9].

**Figure 1:**
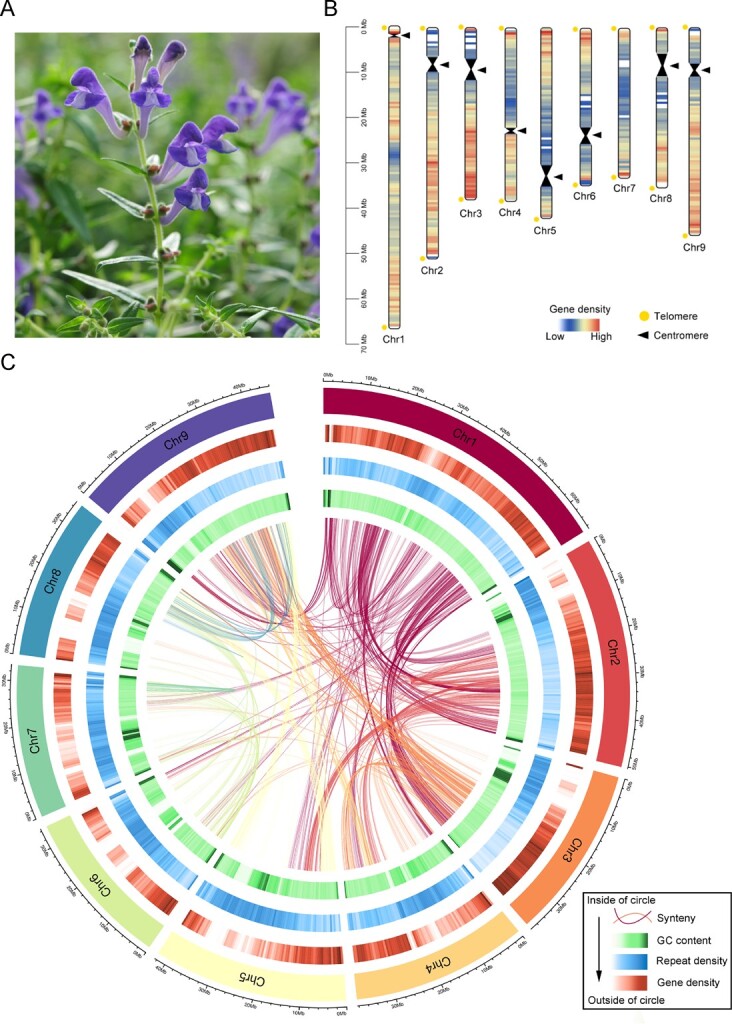
T2T gap-free genome assembly and genomic features of *Scutellaria baicalensis.***A** Image of the *S. baicalensis* plant. **B** Visualization of gene density and the distribution of centromeress and telomere in the *S. baicalensis* genome. The scale represents chromosomes in a 500 kb window. **C** Landscape of the *S. baicalensis* genome assembly. The circles (outer to inner) represent: pseudochromosomes (Chr1 to Chr9), gene density, repetitive sequences density, GC content, and duplicated gene links (3934 gene pairs in total) within the genome. Chromosomes are shown in a 50 kb window.

Anthocyanins belong to a subgroup of flavonoids responsible for the flower coloring, plant biotic and abiotic resistance. In nature, the petals of *S. baicalensis* appear blue to purple owing to the abundant accumulation of delphinidin- and cyanidin-type anthocyanins in the flowers (Fig. 1A) [10]. These two classes of anthocyanins are 3′- or 3′5′- hydroxylated on their B-ring. Anthocyanins are synthesized from the classic flavonoid biosynthetic pathway, which begins with phenylalanine (Fig. S1, see online supplementary material). Phenylalanine is converted to naringenin by phenylalanine ammonialyase (PAL), undergoes ring hydroxylation through cinnamate 4-hydroxylase (C4H), gets activated via 4-coumaryol CoA ligase (4CL), experiences condensation with three molecules of malonyl-CoA by chalcone synthase (CHS), undergoes isomerization by chalcone isomerase (CHI), and is finally hydroxylated through flavanone 3-hydroxylase (F3H), flavonoid 3′-hydroxylase (F3′H), or flavonoid 3′5′-hydroxylase (F3′5′H) to produce dihydrokaempferol, dihydroquercetin, and dihydromyricetin, respectively, which serve as precursors for anthocyanins [11].

Cytochrome P450 (CYP450) is a well-known enzyme superfamily recognized for catalyzing atom monooxygenation to yield compounds like ketones, alcohols, and epoxides [12]. They are named and classified based on sequence similarities and phylogenetic relationships, with a typical CYP450 nomenclature consisting of a family-indicating number followed by a subfamily-defining letter [13]. Plant CYP450s play crucial roles in biosynthesis of specialized metabolites, such as flavonoids (CYP75 and CYP93), terpenoids (CYP76 and CYP706), and alkaloids (CYP80 and CYP719) [14]. Taking advantage of high-quality genome assemblies, more and more genome-wide analyses of the *CYP450* gene family have been undertaken in many plants, including *Oryza sativa* [15], *Glycine max* [16], and *Arabidopsis thaliana* [17]. To date, the functions of over 800 plant *CYP450s* have been elucidated [12]. In *S. baicalensis*, several *CYP450* genes have been functionally characterized, including two genes, *CYP93B24* and *CYP93B25*, encoding flavone synthase II-1 (FNSII-1) and FNSII-2, respectively. FNSII-1 can catalyze both naringenin and pinocembrin to produce apigenin and chrysin, respectively, whereas FNSII-2 can only accept pinocembrin as substrate [5]. In addition, two CYP82D enzymes act as flavone 6-hydroxylase (F6H/CYP82D1.1) and F8H (CYP82D2) which are involved in the synthesis of baicalein and wogonin, respectively [18]. However, genome-wide investigation of the *CYP450* family and genes responsible for the hydroxylation of flavone B-ring have not been studied in *S. baicalensis*.

In 2019, the first reference genome of *S. baicalensis* was sequenced and assembled from a combination of Pacbio, 10× genomics and Hi-C data, with 386.63 Mb of genome size, 1.33 Mb of contig N50, with the sequences anchored into nine pseudochromosomes with a super-N50 of 33.9 Mb [19]. Another genome of *S. baicalensis* was assembled from Oxford Nanopore Technology (ONT) reads with a contig N50 of 2.10 Mb, and the contigs were sorted into nine pseudochromosomes, achieving a super-N50 of 40.8 Mb [20]. Although these assemblies have provided valuable information and helped us identify new genes, they still had hundreds of gaps that obstructed the investigation of complex genomic regions. Taking advantage of newly developed Pacbio HiFi [21] and ONT ultra-long sequencing technologies [22], plant gapless genome assemblies were recently published for rose myrtle (*Rhodomyrtus tomentosa*) [23], kiwifruit (*Actinidia chinensis*) [24] and grapevine (*Vitis vinifera*) [25]. Telomere-to-telomere (T2T) gap-free genome assembly completes the missing sequences, corrects structural errors and enables the accurate analysis of variations among varieties and species, and facilitates the generation of more accurate chromosomal mappings [26].

Here we reported the first T2T gap-free genome of *S. baicalensis* achieved by a integration of PacBio HiFi and ONT ultra-long data, as well as Hi-C technology. In total, 384.59 Mb of genome size was assembled, with all sequences anchored into nine pseudochromosomes, reaching a contig N50 of 42.44 Mb. We determined the major flavonoids accumulated in the flowers. Genome-wide analysis of *CYP450* gene family was performed. Three genes encoding F3′H and one gene encoding F3′5′H were found to be involved in the 3′ or 3′5′ hydroxylation of flavonoids. Our studies give an insight into the formation of blue-purple flowers and provide candidate genes for metabolic engineering of flavonoids.

## Results

### Sequencing and assembly of the *S. baicalensis* T2T gap-free genome

A genome survey revealed that the genome size of *S. baicalensis* was approximately 356.29 Mb, with a heterozygosity rate of 1.08% (Fig. S2, see online supplementary material). A combination of different sequencing platforms was employed to generate a T2T gap-free genome assembly for *S. baicalensis*. We obtained 37.04 Gb Pacbio HiFi reads, 23.51 Gb ONT ultra-long reads and 35.17 Gb Hi-C reads, amounting to ∼103.96×, ∼65.99×, and ∼98.71× coverage of the estimated genome size, respectively (Table S1, see online supplementary material). In order to obtain a high-quality skeleton for T2T genome, three draft assemblies were generated from Pacbio HiFi data and ONT ultra-long data, respectively, as well as a hybrid assembly combining both Pacbio HiFi and ONT ultra-long data (Table S2, see online supplementary material). Among the three assemblies, the one utilizing Pacbio HiFi reads had a size of 397.36 Mb, a contig N50 of 46.00 Mb, and 98.4% completeness, which outperformed the other two draft assemblies and was therefore chosen as the T2T genome skeleton. After removing haplotigs, contaminated and redundant sequences, the HiFi-based assembly was successfully anchored into nine pseudochromosomes using Hi-C assembly, consistent with previous studies [19, 20].

After filling in all remaining gaps with three draft assemblies and correcting errors with HiFi reads, a final 384.59 Mb gap-free genome of *S. baicalensis* was generated, consisting of nine pseudochromosomes with a contig N50 length of 42.44 Mb (Table 1). Interaction heatmap showed that all contigs could be placed in one of the nine pseudochromosomes with strong signal, further validating the high-quality of the genome assembly (Fig. S3, see online supplementary material).

**Table 1:** Summary of T2T gap-free genome assembly and annotations of *Scutellaria baicalensis*.

**Genome assembly**	**Statistics**
Genome size	384.59 Mb
Contig N50	42.44 Mb
Pseudochromosomes	9
Scaffold numbers	9
Hi-C anchoring rate	100%
Gap number	0
GC content	34.6%
Repetitive sequences	65.11%
BUSCO completeness	98.4%
Quality value (QV)	43.60
Structure genes	28 097

### Telomeres and centromeres identifications

By using telomere repeats as queries, we were able to identified 17 telomeres located on the ends of the nine pseudochromosomes (Fig. 1B), the number of motif repeats varied from a minimum of 111 to a maximum of 2673 (Table S3, see online supplementary material). With the exception of Chr5, which only had two motif repeats on its upstream end and therefore was not considered a telomere, every other chromosome had two telomeres located at both ends. (Table S3, see online supplementary material). To predict the putative centromeres regions on the chromosome of *S. baicalensis*, we used short tandem repeats. By combining the Hi-C interaction heatmap with large blank regions (Fig. S3, see online supplementary material), low gene density regions and high LTR/Gypsy density regions, we successfully identified a potential centromere for each chromosome except for Chr7. The length of these putative centromeres ranged from 0.81 Mb to 4.95 Mb (Table S4, see online supplementary material). On Chr7, there were two regions where it was challenging to determine the true centromere (Fig. S4, see online supplementary material). However, further confirmation of the authentic centromere locations will require additional experiments such as fluorescence in situ hybridization (FISH) and chromatin immunoprecipitation (ChIP-seq).

### Structural variations in T2T gap-free genome

By comparing with the previous genome version, various structural variations (SVs), including inversions, translocations, and duplications, were observed across all contigs of the gap-free genome, suggesting an improved quality for the new assembly (Fig. S5A, see online supplementary material). In these SVs, duplication variations accounted for the largest proportion, reaching 89% (Fig. S5B, see online supplementary material). They were mainly concentrated in the telomere and centromere regions, followed by deletion variations and insertion variations, accounting for 14.9% and 12.7%, respectively. Gene Ontology (GO) functional and Kyoto Encyclopedia of Genes and Genomes (KEGG) pathway enrichment analysis of these SV sequences revealed that most sequences were enriched in defense response (Fig. S5C, see online supplementary material), such as plant-pathogen interactions and MAPK signaling pathways (Fig. S5D, see online supplementary material). Furthermore, we used presence and absence variation (PAV) analysis to classify the newly added fragments. GO functional and KEGG pathway enrichment analysis found that most fragments were also enriched in defense response, corresponding to plant hormone signal transduction in KEGG pathway (Fig. S6, see online supplementary material). These results highlight the significance of high-quality genome assembly in plant research.

Multiple indices and methods were employed to assess the assembly’s contiguity, completeness, and accuracy. There was no gap and mismatch (N) in any of the chromosomes, and the count of contigs matched the number of chromosomes (Table S5, see online supplementary material), suggesting a perfect contiguity of the genome assembly. For genome completeness, 99.70%, 99.11%, and 99.98% of Illumina short reads, ONT ultra-long reads, and HiFi reads could be aligned to the assemblies, respectively, covering 99.59%, 99.85%, and 99.77% of the regions on the assembly (Table S6, see online supplementary material). Benchmarking Universal Single Copy Orthologs (BUSCOs) analysis demonstrated that 98.4% (1589 of 1614) of core conserved plant gene orthologs were completely detected in *S. baicalensis* (Table S7, see online supplementary material), indicating a high level of integrity in the genome assembly. The consensus quality value (QV) obtained by comparing the *K*-mer spectrum of short sequencing reads with the assembly indicated a high level of quality and accuracy in the genome assembly (Tables S8 andS9, see online supplementary material).

### Genome annotation

Mixed RNA samples from roots, stems, leaves, and flowers of *S. baicalensis* were sequenced using the Nanopore platform to obtain full-length transcriptome sequences (Table S1, see online supplementary material). Furthermore, Illumina RNA-seq datasets of various tissues were retrieved from NCBI [19]. These RNA sequencing databases were used for genome annotation. The final annotation yielded a total of 28 097 genes. These genes exhibited an average length of 4004 bp and an average coding sequence length of 1161 bp (Table S10, see online supplementary material). BUSCOs analysis showed that 98.2% (1585 of 1614) of the core conserved plant gene orthologs were completely detected in *S. baicalensis* (Table S11, see online supplementary material), indicating a high-confidence annotation of these genes. A total of 26 416 (94.02%) gene products were successfully annotated by at least one of the databases (Table S12, see online supplementary material). The repeat sequence annotation revealed that the *S. baicalensis* genome contained 65.11% repetitive sequences (Table S13, see online supplementary material). Among these, tandem repeats (satellites and microsatellites) made up 0.05%, while interspersed repeats accounted for 4.37%. The most abundant interspersed repeats were long terminal repeats (LTRs) of retroelements, comprising 26.08% of the genome, including 6.32% *Gypsy* LTRs and 9.13% *Copia* LTRs. DNA transposable elements accounted for 33.13%.

The annotation of noncoding RNAs showed that the *S. baicalensis* genome encompassed 115 microRNAs (miRNAs), 434 transfer RNAs (tRNAs), 14 643 ribosomal RNAs (rRNAs), and 580 small nuclear RNAs (snRNAs) (Table S14, see online supplementary material). The integrated distributions of genes, repetitive sequences, GC content, and detected segmental duplications are depicted in Fig. 1C.

### Comparative genomic analysis

**Figure 2:**
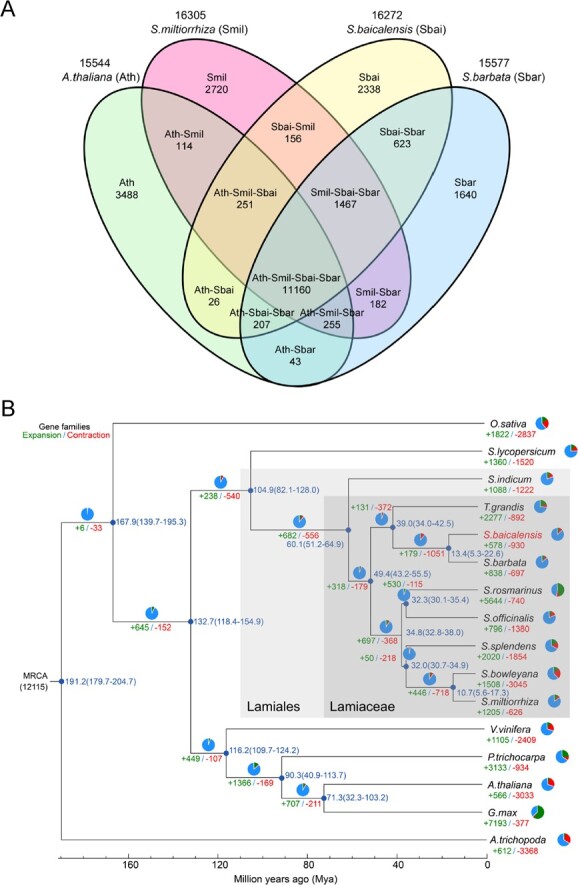
Comparative genomic analysis. **A** Venn diagram displaying shared and unique gene families in *Scutellaria baicalensis* compared to other species. **B** Phylogenetic analysis, estimation of divergence times, and identification of gene family expansions and contractions in *S. baicalensis*. Divergence times (in million years ago, Mya) are indicated by numbers adjacent to each node with confidence intervals (*P* < 0.05) shown in brackets. Gene family contractions and expansions are denoted by negative and positive numbers, respectively.

To elucidate the evolutionary traits and gene families, the genome of *S. baicalensis* was compared with 14 previously published plant genomes, with *Amborella trichopoda*, a basal angiosperm, serving as an outgroup. The gene family clustering analysis pinpointed 16 272 gene families, with 7035 being ubiquitous across all species. Among these shared families, 100 were single-copy gene families (Fig. S7, see online supplementary material). A comparison was made between *S. baicalensis*, two Lamiaceae species (*Salvia miltiorrhiza* and *Scutellaria barbata*), and *A. thaliana*. As depicted in Fig. 2A, 11 160 gene families were found to be shared by *S. baicalensis*, *S. miltiorrhiza*, *S. barbata*, and *A. thaliana*, while 2338 gene families were unique to *S. baicalensis*. Compared with the most recent common ancestor (MRCA) of the 16 plant species, 578 gene families with 2148 genes had expanded, while 930 gene families with 1206 genes had contracted in *S. baicalensis* (Fig. 2B). GO analysis demonstrated that the expanded genes were enriched in pathways related to ‘*O*-methyltransferase activity’ and ‘monooxygenase activity’ (Fig. S8, see online supplementary material), suggesting that gene family expansion played a role in modifying specialized metabolite biosynthesis in *S. baicalensis*, and may explain the prevalence of *O*-methylated and hydroxylated flavones in *S. baicalensis*, which are catalyzed by *O*-methyltransferases and CYP450 monooxygenases [8, 18].

A phylogenetic tree was built from a super-alignment matrix of 290 single-copy orthologous genes across the 16 species. Notably, *Solanum lycopersicum* (Solanales) diverged from Lamiales around 104.9 million years ago (Mya), and *Sesamum indicum* (Pedaliaceae) then diverged from the Lamiaceae around 60.1 Mya. This was followed by the divergence of *Scutellaria* from *Salvia* and *Tectona* about 49.4 and 39.0 Mya, respectively (Fig. 2B). These findings support the previously proposed phylogenetic order [27].

### Metabolomic analysis of flavonoids in the flowers of *S. baicalensis*

**Figure 3:**
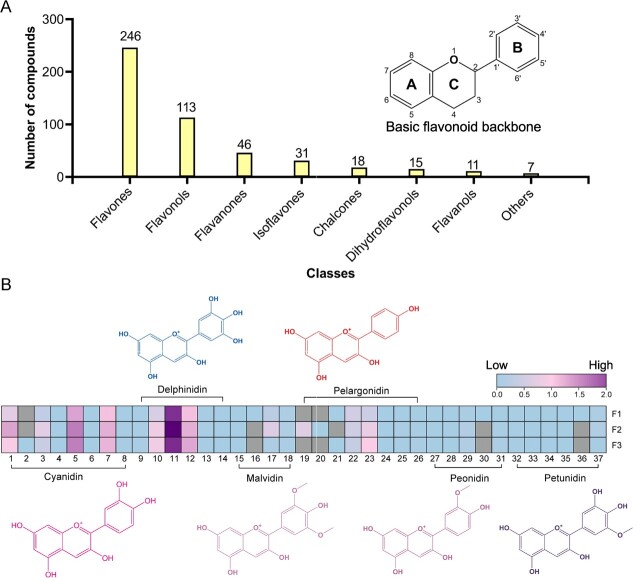
Widely targeted metbolomic analysis of *Scutellaria baicalensis* flowers. **A** Different classes and of flavonoids detected in *S. baicalensis* flowers. **B** Six major classes of anthocyanins, including cyanidin- (Cy), delphinidin- (Dp), malvidin- (Ma), pelargonidin- (Pg), peonidin- (Pn), and petunidin-type (Pt), as well as their content levels detected in *S. baicalensis* flowers: Cy-3,5-*O*-diglucoside (**1**), Cy-3,5,3′-*O*-triglucoside (**2**), Cy-3-*O*-(6-*O*-p-coumaroyl)-glucoside (**3**), Cy-3-*O*-arabinoside (**4**), Cy-3-*O*-(6′′-*O*-malonyl-glucoside) (**5**), Cy-3-*O*-sophoroside (**6**), Cy-3-*O*-glucoside (**7**), Cy-3-*O*-xyloside (**8**), Dp-3-*O*-(6-*O*-acetyl)-glucoside (**9**), Dp-3-O-(6-O-malonyl)-glucoside-3′-glucoside (**10**), Dp-3,5-*O*-diglucoside (**11**), Dp-3-*O*-galactoside (**12**), Dp-3-*O*-sambubioside (**13**), Dp (**14**), Ma-3-*O*-(6-*O*-p-coumaroyl)-glucoside (**15**), Ma-3-*O*-5-*O*-(6-*O*-coumaroyl)-diglucoside (**16**), Ma-3,5-*O*-diglucoside (**17**), Ma-3-*O*-arabinoside (**18**), Pg-3-*O*-galactoside (**19**), Pg-3-*O*-sophoroside (**20**), Pg (**21**), Pg-3-*O*-(6-*O*-p-coumaroyl)-glucoside (**22**), Pg-3-*O*-(6-*O*-malonyl -glucoside) (**23**), Pg-3-*O*-5-*O*-(6-*O*-coumaroyl)-diglucoside (**24**), Pg-3,5-*O*-diglucoside (**25**), Pg-3-*O*-glucoside (**26**), Pn-3-*O*-(6-*O*-p-coumaroyl)-glucoside (**27**), Pn-3-*O*-glucoside (**28**), Pn-3,5-*O*-diglucoside (**29**), Pn-3-*O*-5-*O*-(6-*O*-coumaroyl)-diglucoside (**30**), Pn-3-*O*-(6-*O*-malonyl-glucoside) (**31**), Pt-3-*O*-(6-*O*-malonyl -glucoside) (**32**), Pt-3-*O*-glucoside (**33**), Pt-3-*O*-arabinoside (**34**), Pt-3-*O*-sambubioside-5-*O*-glucoside (**35**), Pt-3-*O*-(6-*O*-p-coumaroyl)-glucoside (**36**), Pt-3-*O*-galactoside (**37**). The content levels of anthocyanins were normalized by log_10_. F, flowers, the numbers 1 to 3 correspond to three biological replicates.

Flavonoids accumulation patterns were assayed using widely targeted metabolome approach [28]. The results revealed the presence of 498 flavonoids found in *S. baicalensis* flowers, with 246 flavones, 113 flavonols, 46 flavanones, 31 isoflavones, 18 chalcones, 15 dihydroflavonols, 11 flavanols, and seven other type flavonoids (Fig. 3A; Table S15, see online supplementary material). Most of these compounds were glycosylated, with their aglycones typically being 3′ or 3′5′ hydroxylated, including eriodictyol, luteolin, quercetin, and myricetin. To further analyse the flower coloring, an anthocyanin-based widely targeted metabolome study was used to quantify the anthocyanins in *S. baicalensis* flower. In total, 40 of 93 targeted anthocyanin compounds were detected, including delphinidin, malvidin, pelargonidin, peonidin and their glycosides, as well as glycosylated cyanidin and petunidin (Fig. 3B; Table S16, see online supplementary material). Cyanidin- and delphinidin-type were two predominant anthocyanins accumulated in the flowers of *S. baicalensis*, such as delphinidin 3,5-*O*-diglucoside (compound 11) and cyanidin 3-*O*-(6′′-*O*-malonyl)-glucoside (compound 5), they reached to the highest content of 176.99 and 33.76 ng/g, respectively (Table S16, see online supplementary material).

### Identification of *flavonoid B-ring hydroxylase* (*FBH*) genes in *S. baicalensis*

**Figure 4:**
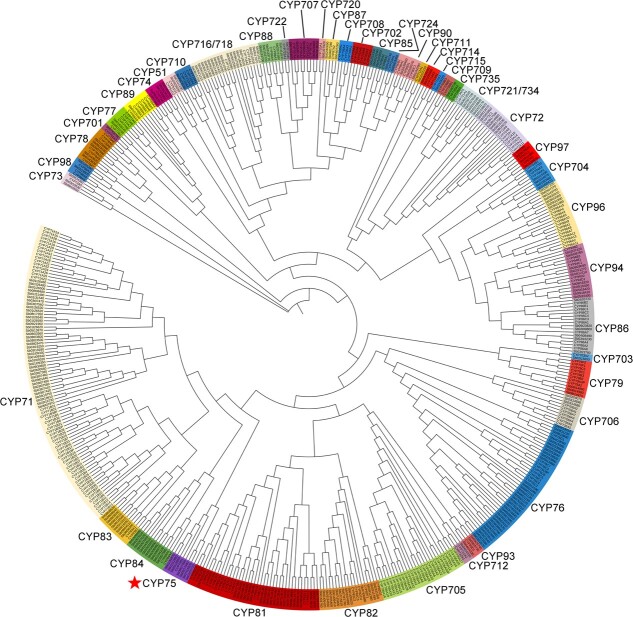
Phylogenetic tree of CYP450s from *Scutellaria baicalensis* and *Arabidopsis thaliana.* Subfamilies were annotated using CYP450s from *A. thaliana*. The phylogenetic tree was constructed using the neighbor-joining (NJ) method with a bootstrap test (*n* = 1000 replications). The star indicates the CYP75 enzymes for further study.

Through a hidden Markov model (HMM) search against the *S. baicalensis* gap-free genome, we extracted 260 full-length CYP450 enzymes. The associated sequences and gene loci details are listed in Table S17 (see online supplementary material). The phylogenetic analysis of these enzymes with CYP450 from *A. thaliana* enabled us to cluster them into 44 subfamilies (Fig. 4). In order to characterize the relative positions of genes, we examined gene clusters in which two or more genes were located within a distance of eight open reading frames (ORFs) on the same chromosome [29]. The chromosomal locations showed that 49 gene clusters containing 60% of all *CYP450* genes (156 of 260) distributed in chromosomes in *S. baicalensis* (Fig. S9, see online supplementary material).

**Figure 5:**
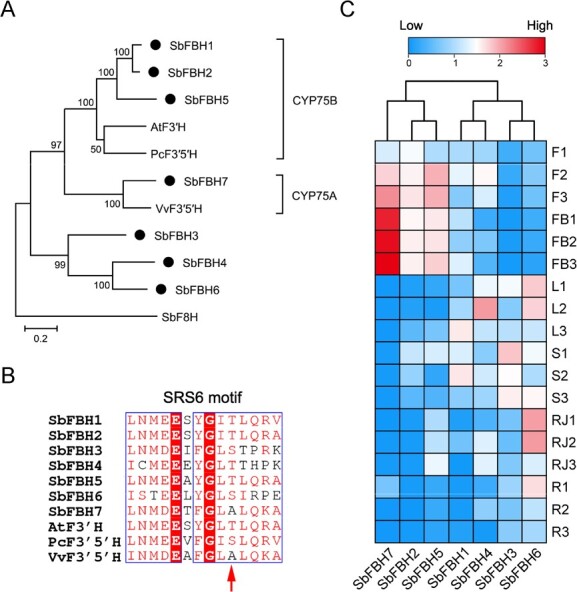
Phylogenetic analysis, multiple sequences alignment and expression patterns of *SbFBH* genes. **A** Phylogenetic tree of SbFBHs and F3′H/F3′5′H from other species. AtF3′H, F3′H from *A. thaliana*, PcF3′5′H and VvF3′5′H, F3′5′H from *Pericallis cruenta* and *Vitis vinifera*, respectively. Flavone 8-hydroxylase from *Scutellaria baicalensis* (SbF8H) was used as an outgroup. **B** The alignment of the SRS6 motif between SbFBHs and F3′H/F3′5′H from different species, with the arrow representing the key amino acid in determination of hydroxylated function **C** Heatmap illustrating the tissue-specific transcripts of *SbFBH* genes. The transcriptional levels are shown as FPKM values normalized by log_10_. F, flower; FB, flower bud; L, leaf; S, stem; RJ, root treated with MeJA; R, root; the numbers 1 to 3 correspond to three biological replicates.

Based on the phylogenetic tree, seven flavonoid B-ring hydroxylase (FBH) candidates (SbFBH1–7) belonging to CYP75 subfamily were identified (Fig. 4; Table S18, see online supplementary material) [30]. To analyse their relationships with F3′H and F3′5′H from different species, we generated a phylogenetic tree using SbFBH1–7 along with several reported sequences. The results (Fig. 5A) showed that SbFBH7 clustered with F3′5′H from *V. vinifera* (VvF3′5′H) in CYP75A subfamily (canonical F3′5′H). SbFBH1, 2 and 5 clustered with enzymes from CYP75B subfamily (canonical F3′H), including F3′H from *A. thaliana* (AtF3′H) and F3′5′H from *Pericallis cruenta* (PcF3′5′H), which were the sister group of the CYP75A clade. SbFBH3, 4 and 6 formed an independent subgroup that was clearly separated from the other two groups, suggesting these enzymes might have undergone neofunctionalization in hydroxylation. Amino acid sequences similarity analysis demonstrated that enzymes of CYP75B (SbFBH1, 2 and 5) subfamilies had a low identity (45.58% to 47.10%) comparing with SbFBH7 from CYP75A (Table S19, see online supplementary material). In CYP75A subfamily, SbFBH1 and 2 had a high identity (91.36%) in sequences but only 67.11% and 67.88% of identity to SbFBH5, respectively. In addition, *SbFBH1* (*Sb01t31870*) and *SbFBH2* (*Sb01t31890*) located adjacently on pseudochromosome 1 (Fig. S9, see online supplementary material), indicating they were tandem duplicated genes with similar functions.

Multiple sequences alignment showed that all SbFBHs possessed CYP450 conserved domains, including a heme-binding domain, an ExxR motif (K helix) and a proton transfer groove (I helix) (Fig. S10, see online supplementary material). In addition, substrate recognition site 6 (SRS6) was identified as a crucial region determining the substrate specificity of CYP450 enzymes, and the eight position within SRS6 is a threonine (Thr) or serine (Ser) residue that are highly conserved in CYP75B F3′H, respectively. Conversely it is typically an alanine (Ala) residue in F3′5′H of CYP75A [31]. SbFBH1–6 possessed a Thr or Ser residue at the eight position of SRS6 motif, while SbFBH7 has an Ala residue within SRS6 at the same position (Fig. 5B). These findings suggest that SbFBH7 is an F3′5′H, while SbFBH1–6 are F3′H enzymes, potentially responsible for the 3′- or 5′-hydroxylation of flavones in *S. baicalensis*.

Based on fragments per kilobase of exon model per million mapped fragments (FPKM) values obtained from RNA-seq of different tissues in *S. baicalensis*, it was observed that *SbFBH2*, *5* and *7* exhibited comparatively high expression levels in both flowers and flower buds (Fig. 5C), which were likely involved in the pigmentation process of flowers. The average FPKM value of *SbFBH7* in flower buds was 22.16 and 16.63 times higher than those in *SbFBH2* and *5*, respectively (Table S20, see online supplementary material), suggesting that SbFBH7 was the key enzyme involved in the coloration of *S. baicalensis* flowers. Transcripts of *SbFBH1* and *4* were equally accumulated in plant aerial parts, while *SbFBH3* and *6* exhibited high expression levels in leaves, roots, and stems. Additionally, the transcripts of *SbFBH4*, *5*, and *6* were up-regulated in JA-treated roots, indicating their potential involvement in the hydroxylation of flavones in the roots.

### Functional characterization of SbFBHs

**Figure 6:**
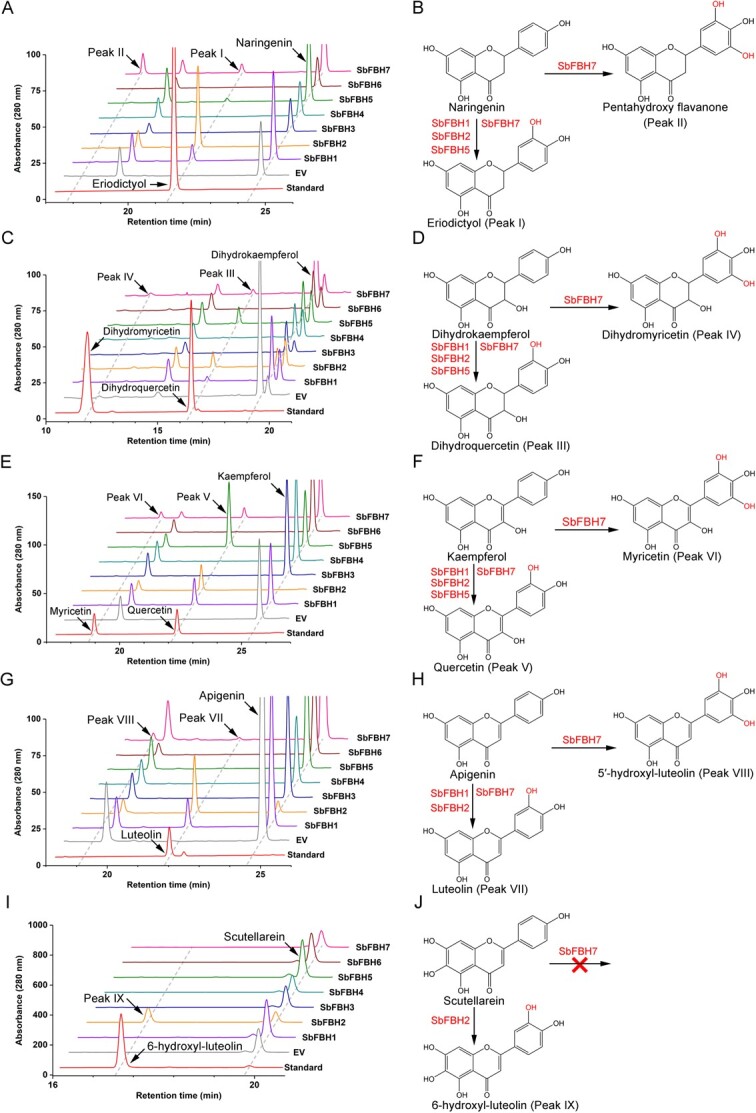
Yeast enzyme assays of SbFBHs. **A**–**J** HPLC analysis and the reaction catalyzed by SbFBHs using naringenin (**A**, **B**), dihydrokaempferol (**C**, **D**), kaempferol (**E**, **F**), apigenin (**G**, **H**), and scutellarein (**I**, **J**) as substrates in yeast enzyme assays, respectively.

To further analyse the enzymatic functions, full-length ORFs of *SbFBHs* were successfully constructed into the expression vectors and transformed into yeast. The strains were then fermented with different substrates. Novel peaks (Peak I) corresponding to the retention time of the eriodictyol standard were detected when SbFBH1, 2, 5, and 7 were incubated with naringenin, in comparison to the empty vector (EV) control (Fig. 6A and B). These products exhibited identical mass charge ratio (m/z) and MS/MS patterns to that of the eriodictyol standard (Fig. S11A, see online supplementary material). Additionally, another new peak (Peak II) was detected from SbFBH7 fed with naringenin (Fig. 6A), which MS spectrum ([M-H]^−^ = 303.0515) was 16 mass units more than eriodictyol ([M-H]^−^ = 287.0563) (Fig. S11A, see online supplementary material). Its MS/MS spectrum was very similar to eriodictyol and the major fragment of MS/MS ([M-H]^−^ = 151.0400) was also 16 more than eriodictyol ([M-H]^−^ = 135.0452), indicating the presence of an additional hydroxyl group compared to eriodictyol. Therefore, we speculated Peak II was 5′- hydroxylation and corresponded to pentahydroxy flavanone (Fig. 6B). When SbFBH1, 2, and 5 were fed with dihydrokaempferol, the substrate was converted to dihydroquercetin (Peak III), while SbFBH7 converted the substrate to both dihydroquercetin and dihydromyricetin (Peak IV) (Fig. 6C and D). These new products possessed the same MS and MS/MS patterns as the dihydroquercetin and dihydromyricetin standards (Fig. S11B, see online supplementary material). Dihydroquercetin and dihydromyricetin are precursors of cyanidin- and delphinidin-type anthocyanins, respectively, which may be responsible for the coloration of *S. baicalensis* flower. These results indicate that SbFBH1, 2, and 5 was F3′Hs and SbFBH7 was an F3′5′H involved in anthocyanins biosynthesis.

Following the same protocol, we also found that SbFBH1, 2, and 5 could hydroxylate the 3′-position of kaempferol to produce quercetin (Peak V) (Fig. 6E and F), while SbFBH7 converted the substrate to both quercetin and myricetin (Peak VI) (Fig. 6E and F). The identification of these products was confirmed through a comparison of their retention time, m/z ratio, and MS/MS patterns with standards (Fig. S11C, see online supplementary material). SbFBH1 and 2 could covert apigenin to luteolin (Peak VII), and SbFBH7 could covert apigenin to 5′-hydroxyl luteolin (Peak VIII, one hydroxyl group more than luteolin determined by MS/MS spectrum) (Fig. 6G and H; Fig. S11D, see online supplementary material). Interestingly, only SbFBH2 demonstrated the capacity to hydroxylate the 3′-position of scutellarein, producing 6-hydroxyl-luteolin (Peak IX), with matching retention time and MS/MS pattern to the standard (Fig. 6I and J; Fig. S11E, see online supplementary material). In addition, none of the SbFBHs could use 4′-deoxyflavonoid as substrates, including pinocembrin, chrysin, and baicalein (Fig. S12, see online supplementary material).

## Discussion

This study provided the first T2T gap-free genome of *S. baicalensis*, with a genome size of 384.59 Mb and a contig N50 of 42.44 Mb. The sequences were successfully anchored into nine pseudochromosomes. Benefits from the development of long-read sequencing technologies, the new genome assembly’s quality has significantly improved compared to the previous two versions [19, 20]. The new assembly has a higher contiguity (0 gap and mismatch, intact contig for each pseudochromosome), completeness (BUSCO 98.4%), and correctness (quality value 43.6). A detailed comparison of three genome versions was presented in Table S21 (see online supplementary material). With the aid of gap-free genome, we identified 260 non-redundant *CYP450* family genes (Fig. 4; Table S17, see online supplementary material), while there were only 205 and 238 *CYP450* genes that could be extracted from the two old genome versions, respectively. For CYP75 subfamily, *SbFBH1* and *5* were missing in the two old genome versions, which highlights the importance of a complete genome in the analysis of biosynthetic pathways for specialized metabolites.

Approximately 300 flavonoids have been identified in *S. baicalensis* [4]. Previous studies have primarily focused on the 4′-deoxyflavones, such as baicalin, wogonoside, baicalein, and wogonin, which are highly accumulated in the roots. These compounds are known to contribute to the majority of the health benefits associated with *S. baicalensis*, including a broad range of antitumor properties [3]. Anthocyanins are an important subgroup of flavonoids possessing antioxidant, anticancer, and antibacterial properties, in addition to serving as flower pigments [32]. Anthocyanins can be classified into six major classes: cyanidin, delphinidin, malvidin, petunidin, pelargonidin, and peonidin, which contribute to a diverse range of colors observed in flowers. For instance, delphinidin-based anthocyanins contribute to blue and purple colors, while cyanidin-based anthocyanins give rise to magenta and red colors. The color spectrum of anthocyanins is influenced by the number of hydroxyl groups present on the B-ring, with greater hydroxylation resulting in bluer color [33]. Metabolome analysis has revealed that purple and purple-red flowers of *S. baicalensis* variants contained significantly higher levels of cyanidin- and delphinidin-type anthocyanins compared to white flowers, while the purple flower accumulated more delphinidin-type anthocyanins but fewer cyanidin-type compared with the purple-red variant [10]. Our flower-specific metabolome analysis showed that delphinidin 3,5-*O*-diglucoside was the highest anthocyanins accumulated in the flower of *S. baicalensis* (Fig. 3B; Table S16, see online supplementary material), consistent with the previous conclusion that delphinine-based anthocyanins are the key compounds conferring blue-purple pigments in flowers [34]. In addition, a metabolome analysis revealed the presence of 487 flavonoids in the flowers of *S. baicalensis* (Fig. 3A; Table S15, see online supplementary material), and these compounds were thought to be co-pigments requiring for the blue flower, such as chalcone, apigenin (flavone), and kaempferol (flavonol), which are also involved in the attraction of insects [35].

In *S. baicalensis*, two separate flavonoid biosynthetic pathways have been identified (Fig. S1, see online supplementary material) [6]. The synthesis of 4′-deoxyflavones originates from a newly evolved root pathway that uses cinnamate-CoA ligase (CLL-7), a specific isoform of chalcone synthase (CHS-2), a conserved chalcone isomerase (CHI), and a specialized isoform of flavone synthase II-2 (FNSII-2) to produce chrysin as the precursor [5]. It emerges after the divergence of the genus *Scutellaria* and *Salvia* following point mutation, tandem multiplication, segmental duplication, and retroduplication [19]. Alternatively, anthocyanins and 4′-hydroxyflavones are synthesized by the canonical pathway that uses naringenin as their precursor. F3′H and F3′5′H are crucial enzymes involved in determining flower color, which catalyze the conversion of naringenin and dihydrokaempferol into dihydroquercetin and dihydromyricetin, respectively. Dihydromyricetin serves as the precursor for the synthesis of delphinidin. Many plant species do not exhibit blue flower color due to the lack of the F3′5 ′H enzyme, which is responsible for the formation of delphinidin-based anthocyanins [33]. Thus, the study of F3′5′H and the mechanism of flavonoid B-ring hydroxylation are important in breeding of ornamental plants and metabolic engineering of blue pigments. The first *F3′H* [36] and *F3′5′H* [37] genes were cloned and functional characterization in *Petunia hybrid*, and since then, these genes have been identified and isolated from numerous plant species. Most F3′H enzymes belong to the CYP75B subfamily, while F3′5′H enzymes are predominantly classified under the CYP74A subfamily [33]. Functional characterization and phylogenetic analysis of F3′H and F3′5′H in the Asteraceae family revealed that F3′5′H evolved from F3′H prior to the divergence of angiosperms and gymnosperms [30]. However, some Asteraceae species lost the *CYP75A* gene in their genome but retained the 3′5′- hydroxylase by duplication and neofunctionalization of an F3′H CYP75B gene, such as *CcF3′5′H* from *Callistephus chinensis*, *OhF3′5′H* from *Osteospermum hybrida* and *PcF3′5′H* from *P. cruenta*, which belong to CYP75B rather than CYP75A [30]. In *S. baicalensis*, SbFBH1, 2, and 5 were clustered into CYP75B subfamily with AtF3′H and PcF3′5′H (Fig. 5A), which demonstrated the ability to hydroxylate the 3′-position of naringenin and dihydrokaempferol, resulting in the production of eriodictyol and dihydroquercetin, respectively (Fig. 6A–D). SbFBH7 was a canonical F3′5′H clustered into CYP75A with VvF3′5′H (Fig. 5A), which exhibited the ability to hydroxylate both the 3′- and 5′-positions of naringenin and dihydrokaempferol, resulting in the production of pentahydroxy flavanone and dihydromyricetin, respectively (Fig. 6A–D). As *SbFBH7* had a much higher expression level than *SbFBH1*, *2*, and *5* in flower buds (Fig. 5C;Table S20, see online supplementary material), which might result in the large accumulation of delphinidin-type anthocyanins in *S. baicalensis* flowers, consistent with our metabolome data (Fig. 3B;Table S16, see online supplementary material). SbFBH3, 4, and 6 were classified into a distinct subfamily within the CYP75 clan; however, they did not demonstrate any activity towards the flavanone, flavanol, dihydroflavonol, flavonol, and flavone substrates we used, so further investigation is required to investigate their functions. In addition, SbFBH1, 2, and 5 could accept more 4′-hydroxyflavonoids as substrates, such as kaempferol (SbFBH1, 2, and 5), apigenin (SbFBH1 and 2), and scutellarein (SbFBH2) to produce quercetin, luteolin, and 6-hydroxyluteolin, respectively, while SbFBH7 could covert kaempferol and apigenin to myricetin and 5′-hydroxyluteolin, respectively (Fig. 6E–J). These products are further glycosylated or methylated to a diversity of compounds that could be detected in *S. baicalensis* flowers (Table S15, see online supplementary material). However, kaempferol and apigenin were mostly glycosylated in *S. baicalensis* flowers (Table S15, see online supplementary material), and only SbFBH2 could accept scutellarein as substrate, which derived flux along the anthocyanins biosynthesis. Interestingly, none of the SbFBHs could use 4′-deoxyflavonoid as their substrates, including pinocembrin, chrysin, and baicalein, consistent with the results of CitF3′H from *Citrus clementina* [14].

Flower color is a crucial characteristic in ornamental plants. However, there are only 15%–20% of wild species with bluish flowers in nature and most of the best-selling ornamental plants lack blue varieties, such as carnation (*Dianthus caryophyllus*), chrysanthemum (*Chrysanthemum morifolium*) and rose (*Rosa hybrida*) [38]. Molecular breeding is an effective way to obtain ornamental plants with various flower colors in floriculture industry. Noda *et al*. had successfully generated blue chrysanthemum flowers by overexpression of a butterfly pea (*Clitoria ternatea*) UDP-glucosyltransferase gene and a *F3′5′H* from *Canterbury bells* [39]. *S. baicalensis* is a potential ornamental plant with numbers of natural blue-purple flowers in a single inflorescence, as well as its long flowering period from end spring to autumn. The highly expressed *F3′5′H* in flower buds of *S. baicalensis* (*SbFBH7*) is also a strong candidate gene in genetic engineering for blue ornamental flowers.

## Conclusions

Here we reported the first gap-free genome of *S. baicalensis*, along with the identification and quantification of major flavonoids in its flowers. Additionally, we performed a thorough analysis of the *CYP450* gene family in *S. baicalensis* and performed functional assays on SbFBHs. These works provided new insights into the hydroxylation of flavonoids B-ring. These findings serve as a basis for breeding blue flower germplasm in horticulture and offer a toolkit for the biosynthesis of blue anthocyanin through synthetic biology.

## Materials and methods

### Plant materials, DNA extraction, library construction and sequencing

All sequencing materials were sourced from an individual *S. baicalensis* plant that was cultivated and maintained at Shanghai Chenshan Botanical Garden. High-quality genomic DNA was obtained from fresh leaves employing a modified cetyltrimethylammonium bromide approach [40]. The integrity and concentration of the DNA were detected through gel electrophoresis, spectrophotometer (ThermoFisher, MA, USA) and Fluorometer (Life Technologies, CA, USA). An ultrasonic disruptor (Covaris, MA, USA) was utilized to randomly shear the DNA. The library of Illumina sequencing was constructed following the sample preparation guide (#15026486, Illumina, CA, USA) and sequenced on Illumina NovaSeq 6000 with 150 bp pair-ends.

For ONT ultra-long sequencing, the library was built using the Ligation Sequencing Kit (#SQK-LSK110, ONT, Oxford, UK) with the standard protocol. The purified library was loaded onto Flow Cells and sequenced on the Nanopore PromethION sequencer.

For PacBio HiFi sequencing, SMRT bell libraries with 15 kb insert fragments were constructed using Biosciences SMRT bell express template prep kit 2.0 (Pacific Biosciences, CA, USA) with the standard protocol. The library was sequenced on PacBio sequel II sequencing platform with 30 h.

For Hi-C sequencing, chromatin was fixed using formaldehyde. The DNase-based protocol was employed to capture *in situ* Hi-C chromosome conformation [41]. The library was sequenced on an Illumina NovaSeq with 150 bp paired-ends mode.

For full-length transcriptome sequencing, RNA from roots, stems, leaves, and flowers were extracted using Plant RNA Kit (#R6827, OMEGA, GA, USA) with the standard protocol. The mRNA was enriched to synthesis cDNA following the strand-switching protocol from Oxford Nanopore Technologies. Then the PCR-amplified cDNA was sequenced on a Nanopore PromethION sequencer.

### Genome survey

To obtain clean data, Illumina DNA sequencing data was filtered to remove low quality sequences and adapter sequences using fastp v0.21.0 [42]. *K*-mer based analysis was employed to estimate genome size and heterozygous ratio with Jellyfish v2.2.10 and GCE v1.0.0 [43]. To determine whether the sequencing data was contaminated, the first 50 000 reads were extracted and compared with Nucleotide Sequence Database (NT, 202107 version) using blastn v2.11.0+, and the species classification was performed using MEGAN v6.16.4 [44]. The parameters of software used in this study were detailed in Table S22 (see online supplementary material).

### T2T gap-free genome assembling, hi-C anchoring and telomere repair

After removal of short reads (<10 kb) and adaptor sequences using Filtlong v0.2.4 and Porechop v0.2.4, resepectively, ONT ultra-long reads were preliminarily assembled using NextDenovo v2.5.0. The ONT draft genome was error-corrected using Racon v1.4.11 with ONT ultra-long sequencing data and Pilon v1.23 with Illumina sequencing data. For Pacbio HiFi draft genome assembly, CCS v6.0.0 was used to remove low-quality reads, and then assembled using hifiasm v0.16.1-r375. In addition, a hybrid assembly using both ONT ultra-long and Pacbio HiFi data was obtained using hifiasm v0.18.2-r467.

After removing haplotigs using Purge_dups v1.2.5 [45], as well as plastidial and bacterial sequence contaminants using Minimap2 v2.17-r941 [46], the HiFi-based assembly was further anchored into pseudochromosomes using Hi-C technology. The raw Hi-C sequencing data was filtered using fastp v0.21.0 to obtain clean data [42]. The clean data was mapped onto the genome assembly by HICUP v0.8.0 to remove unmapped reads, invalid pairs, and repeats. Successively, contigs were clustered, ordered, and orientated by ALLHiC v0.9.8 [47]. 3D-DNA v180419 [48], Juicer v1.6 [49], and Juicebox v1.11.08 [50] were used to order and orientate the contigs manually. Finally, redundant contigs were removed manually based on the interaction relationship, and 100 Ns was used to fill the gap. The interaction heatmap was plotted with HiCExplorer v3.6 [51].

To obtain T2T gap-free genome assembly, ONT ultra-long reads was mapped to the genome assembly to collect the reads on the terminal of each pseudochromosome with a 50 bp screening window using Winnowmap v1.11 [52]. The numbers of telomere repeats (CCCATTT at the 5′ end and TTTAGGG at the 3′ end) were searched for all reads and the read with the most counts was labeled as reference, with others as queries. These reference and query sequences were assembled using medaka_consensu v1.2.1. These consensus sequences then replaced the terminal sequences on each pseudochromosome using nucmer v3.1 [53]. The T2T gap-free genome assembly underwent an error correction using Racon v1.6.0 with Pacbio HiFi reads. Centromere locations were predicted using Tandem Repeat Finder (TRF) with 60 kb screening window.

To compare the differences between the T2T gap-free assembly and the old version of the genome, MUMmer v4.0.0rc1 was used to conduct whole-genome alignment, and SyRI v1.6 was used to detect structural variations. The parameters of software used in this study were detailed in Table S22 (see online supplementary material).

### Genome quality assessment

The continuity of the genome was assessed by determining the number of contigs, N50 length, and the number of gaps. The genome completeness was assessed using BUSCO v5.3.0. To assessment of genome correctness, ONT ultra-long reads and Pacbio HiFi reads were aligned to genome assembly for calculating the mapping rate using BWA v0.7.17-r1188 and minimap2 v2.17-r941, respectively. The quality and accuracy of the genome assembly were evaluated using the consensus quality value (QV), which involved comparing the *K*-mer spectrum of Illumina sequencing reads with the assembled genome. A comparison of the gap-free assembly with the old version genome was performed using mummer v4.0.0 and then identified the syntenic relationship and structural variants by syri v1.6. The parameters of software used in this study were detailed in Table S22 (see online supplementary material).

### Genome annotation

Repetitive sequences were identified by homolog alignment and *de novo* prediction. RepeatModeler v1.0.11 and LTR_FINDER (Official release of LTR_FINDER_parallel) were used to build a *de novo* repetitive element database. LTR_retriever v2.9.0 was used to remove the redundant LTR sequences. For homolog alignment, the *de novo* repetitive element database and Repbase database (2 018 026 version, http://www.girinst.org/repbase) employing RepeatMasker v4.0.9 was used to predict repetitive sequences. RepeatProteinMask v4.0.9 was used to predict TE proteins. After removal of redundant sequences, the final repetitive sequence set was obtained.

Gene structure was annotated using a combined strategy including homologous prediction, *de novo* gene prediction, as well as gene prediction based on RNA-seq and PacBio data. For homologous prediction, protein sequences were mapped to the reference genome, including *A. thaliana*, *S. barbata*, *Salvia splendens*, and *S. miltiorrhiza* using TblastN v2.7.1 [54], then Exonerate v2.4.0 was used to predict the transcripts and coding region [55]. In addition, genes predicted by BUSCO (which was performed in genome quality assessment) were also used as the homolog prediction results [56]. *De novo* gene structure identification was based on Augustus v3.3.2 [57] and GlimmerHMM v3.0.4 [58]. RNA-seq reads were filtered using fastp v0.21.0 [42], and were aligned to the genome using HISAT2 v2.1.0 [59]. Alignment results were then used as input for Stringtie v2.1.4 to obtain transcripts [60], and then predicted using TransDecoder v5.1.0. Nanopore RNA-seq reads were filtered using NanoFilt v2.8.0. Full-length sequences were identified using Pychopper v2.7.2. After error correction using racon v1.4.21, these full-length sequences were aligned to genome using minimap2 v2.17-r941 [61]. Alignment results were then used as input for Stringtie v2.1.4 [60], and then predicted using TransDecoder v5.1.0. All predicted gene sets were then merged into a gene set via MAKER v2.31.10 [62], which underwent further refinement to derive the final gene set. The completeness of this genome annotation was validated using BUSCO v5.3.0.

The gene functions of the finalized gene set were determined by conducting protein sequence comparisons against multiple well-known databases, including Universal Protein (Uniprot) v202011 [63], Non-Redundant Protein Sequence Database (NR) v202011 [64], and KEGG [65] with DIAMOND blastp v2.0.11.149 [66]. KOBAS v3.0 was used to enrich the KEGG orthology and pathway [67]. GO IDs were assigned from the Unipro database, while motifs and domains were annotated through InterProScan v5.52–86.0 [68] and Protein Families Database (Pfam) v202011 with hmmscan v3.3.2 [69].

Noncoding RNA structures were annotated via tRNAscan-SE v1.23 for tRNA [70] and INFERNAL v1.1.2 for ncRNA, miRNA and snRNA [71]. rRNAs were predicted using the RNA families (Rfam) database (https://rfam.org/). The parameters of software used in this study were detailed in Table S22 (see online supplementary material).

### Comparative genomic analysis

A gene family clustering was performed for 16 plant species, which comprised *S. baicalensis*, *A. trichopoda*, *A. thaliana*, *G. max*, *O. sativa*, *Populus trichocarpa*, *Salvia bowleyana*, *S. miltiorrhiza*, *Salvia officinalis*, *Salvia rosmarinus*, *S. splendens*, *S. barbata*, *S. indicum*, *S. lycopersicum*, *Tectona grandis*, and *V. vinifera*, utilizing blastp v2.6.0 [54] and Orthofinder v2.3.12 [72]. These species were used to extract single-copy orthologous genes, which were then aligned using MUSCLE v3.8.31. [73]. Alignment results were filtered using trimal v1.2rev59 [74] and combined to create a super-alignment matrix. A maximum likelihood (ML) phylogenetic tree was built using RAxML v8.2.10 with the PROTGAMMAWAG model [75]. To estimate the divergence times between species, the MCMCtree v4.9 program from PAML [76] was employed. The following calibration points extracted from TimeTree [77] were applied: *T. grandis*–*S. barbata* (19.4–39.9 Mya), *S. miltiorrhiza*–*S. splendens* (30.9–35.8 Mya), *S. officinalis*–*S. indicum* (32.6–63.0 Mya), *V. vinifera*–*A. thaliana* (109.8–124.4 Mya), and *O. sativa*–*A. trichopoda* (179.9–204.9 Mya). Gene family expansions and contractions were identified via CAFÉ v3.1 [78], followed by enrichment to GO and KEGG for functional annotation. The parameters of software used in this study were detailed in Table S22 (see online supplementary material).

### Widely targeted metabolomic analysis

For flavonoid metabolome, flowers with three biological replicates were collected, freeze-dried, and ground into powder. A total of 50 mg of the powdered samples were suspended in 1200 μl of pre-cooled 70% (v/v) methanol, and then extracted for 6 h, with a vortex mixing every 30 min for 30 s. The samples were centrifuged at 12000 rpm for 3 minutes to collect the supernatant and filtered with a 0.22 μm membrane filter before UPLC–MS/MS analysis. Samples were analysed using a UPLC-ESI-MS/MS system (UPLC, ExionLC™AD; MS, Applied Biosystems 4500 Q TRAP). Chromatographic separation was carried out on an Agilent SB-C18 column (1.8 μm, 2.1 mm × 100 mm). The mobile phase comprised 0.1% (v/v) formic acid in pure water (A) and 0.1% (v/v) formic acid (v/v) with acetonitrile (B). The gradient program started with 95% A, followed by a linear gradient to 5% A within 9 minutes, and maintaining 5% A for 1 minute. Then, the composition was set back to 95% A within 1.1 minutes and held for 2.9 minutes. The HPLC system operated at a flow rate of 0.35 ml/min, while the column oven maintained a temperature of 40°C. A 4 μl injection volume was used for sample introduction. The ESI source temperature was set to 550°C, and the ion spray (IS) voltage was set at 5500 V/−4500 V in negative mode. The ion source gas I, gas II, and curtain gas pressures were set to 50, 60, and 25 psi, respectively. For fragmentation, the collision-activated dissociation (CAD) level was set to high. The acquisition mode employed was multiple reaction monitoring (MRM) using medium nitrogen collision gas in triple quadrupole (QQQ) scans.

For anthocyanin quantify metabolome, flowers with three biological replicates were collected, freeze-dried, and ground into powder. 50 mg of the powdered samples were suspended in 500 μl of 500:500:1 methanol/water/hydrochloric acid and extracted using vortex mixing and ultrasound for 5 min each. The samples were then centrifuged at 4°C, 12000 rpm for 3 min. The supernatant was collected, and the residue was re-extracted. The supernatant was combined and filtered through a 0.22 μm membrane filter before UPLC–MS/MS analysis. The UPLC–MS/MS analysis was performed using an UPLC system (ExionLC™AD) coupled with a QQQ (Applied Biosystems 6500). The chromatographic separation was conducted using a Waters ACQUITY BEH C18 column (1.7 μm, 2.1 mm × 100 mm). The mobile phase consisted of 0.1% formic acid in pure water (A) and a mixture of 0.1% formic acid with methanol (B). A gradient program was employed, starting with 95% A, transitioning to 50% A at 6 minutes, reaching 5% A at 12 minutes, and returning to 95% A at 14 minutes. The flow rate was set at 0.35 ml/min, and the column temperature was maintained at 40°C. A 2 μl injection volume was used. The mass spectrometer operated in the ESI+ mode, with the source temperature set at 550°C. The IS voltage was 5500 V, and the curtain gas pressure was set at 35 psi. Anthocyanins were analysed using MRM mode. The quantification of anthocyanin levels was performed by comparing the peak areas with standard curves generated from standard compounds.

### Genome-wide identification and phylogenetic analysis of *CYP450* genes

To identify full-length *CYP450* candidates in the *S. baicalensis* genome, the hidden Markov model (HMM) profile of PF06200 was utilized. The HMM algorithm (HMMER) [79] was applied for extraction, and candidates with a length <400 and >600 amino acids were filtered [80]. Multiple sequence alignments and the construction of phylogenetic trees were carried out using MEGA X [81]. For the neighbor-joining tree, CYP450 sequences from *A. thaliana* were included. The maximum-likelihood tree was constructed with SbFBHs sequences and previously reported F3′H and F3′5′H under the following accession numbers: AtF3′H (NP_196416), PcF3′5′H (ABB43030), and VvF3′5′H (NP_001268157.1).

### Gene cloning and yeast expression vector construction

The full-length ORFs of *SbFBH* genes were amplified using gene-specific primers listed in Table S18 (see online supplementary material). *SbFBH1* and *SbFBH5* were synthesized *de novo* (GenScript, Nanjing, China). The fragments were cloned into the entry vector pDONR207 and the yeast expression vector pYesdest52 using the Gateway BP and LR Clonase II Enzyme Kit (Invitrogen, MA, USA), respectively.

### *In vivo* yeast enzyme assays

The yeast expression vector constructs or an empty vector were introduced into the yeast strain WAT11 [82, 83] and screened on SD-Ura medium with 20 g/L glucose. Following incubation at 28°C for 48–72 hours, transformant colonies were initially cultured in 20 ml of SD-Ura liquid medium supplemented with 20 g/L glucose at 28°C until reaching an OD_600_ of 2–3. The yeast cells were subsequently harvested by centrifugation at 4000 *g*, followed by re-suspension in 20 mL of SD-Ura liquid medium supplemented with 20 g/L galactose to induce the expression of target proteins. Different flavonoid substrates were added to the cultures at a final concentration of 50 μM. After 48 h of fermentation (with an additional 2 mL of galactose added after 24 h), yeast cells were collected by centrifugation at 12000 rpm for 5 min, and extracted with 1 mL of 70% methanol (pH 5.0) using an ultrasonic water bath for 3 h. The supernatants were filtered through a 0.22 μm membrane filter and subsequently analysed by HPLC and LC–MS.

Standard compounds of naringenin, eriodictyol, apigenin, luteolin, scutellarein, kaempferol, pinocembrin, chrysin, and baicalein were purchased from Sigma-Aldrich (MO, USA). Standard compounds of quercetin, myricetin, dihydrokaempferol, dihydroquercetin, dihydromyricetin, and 6-hydroxyluteolin were purchased from Yuanye-Biotech (Shanghai, China). All of the above standard compounds were dissolved in dimethyl sulfoxide (DMSO).

### Metabolite analyses

Metabolite analysis was conducted using HPLC (Agilent 1260 Infinity II). A C18 column (Phenomenex Luna, 100 mm × 2 mm, 3 μ) was employed for chromatographic separation. The mobile phase, consisting of 0.1% formic acid in pure water (A) and a mixture of acetonitrile/methanol (1:1) with 0.1% formic acid (B), was delivered at a flow rate of 0.26 ml/min. The gradient program was as follows: 0–3 min, 80% A; 20 min, 50% A; 20–30 min, 50% A; 36 min, 70% A; 37 min, 80% A; and 37–43 min, 80% A. Detection was performed at a wavelength of 280 nm. The injection volume was 20 μl, and the column temperature was maintained at 35°C. Mass spectra were acquired using Thermo Q Exactive Plus in the negative ion mode with a heated ESI source. The auxiliary gas flow, auxiliary gas heater, sheath gas flow, spray voltage, and capillary temperature were set as 10 l/min, 350°C, 40 l/min, 3.5 kV and 20°C, respectively.

## Supplementary Material

Web_Material_uhad235

## Data Availability

Illumina RNA sequencing data of different tissues from *S. baicalensis* are extracted from the Sequence Read Archive (SRA) database (www.ncbi.nlm.nih.gov/sra) with the accession number SRP156996. The two previous versions of *S. baicalensis* genome are available in the National Genomics Data Center (NGDC, https://bigd.big.ac.cn/gwh) with accession number GWHAOTC00000000 and GWHAOTO00000000, respectively. The T2T gap-free genome assembly of *S. baicalensis* is available in NGDC with accession number GWHDEDD00000000.
