# Supplementary material for: Gap-free genome assembly and CYP450 gene family analysis reveal the biosynthesis of anthocyanins in Scutellaria baicalensis
Source: Hortic Res. 2023 Nov 17;10(12):uhad235. doi: 10.1093/hr/uhad235 (PMC10753160; doi:10.1093/hr/uhad235)
Supplement: Web_Material_uhad235 [file web_material_uhad235.zip › Supplementary Figures.docx]

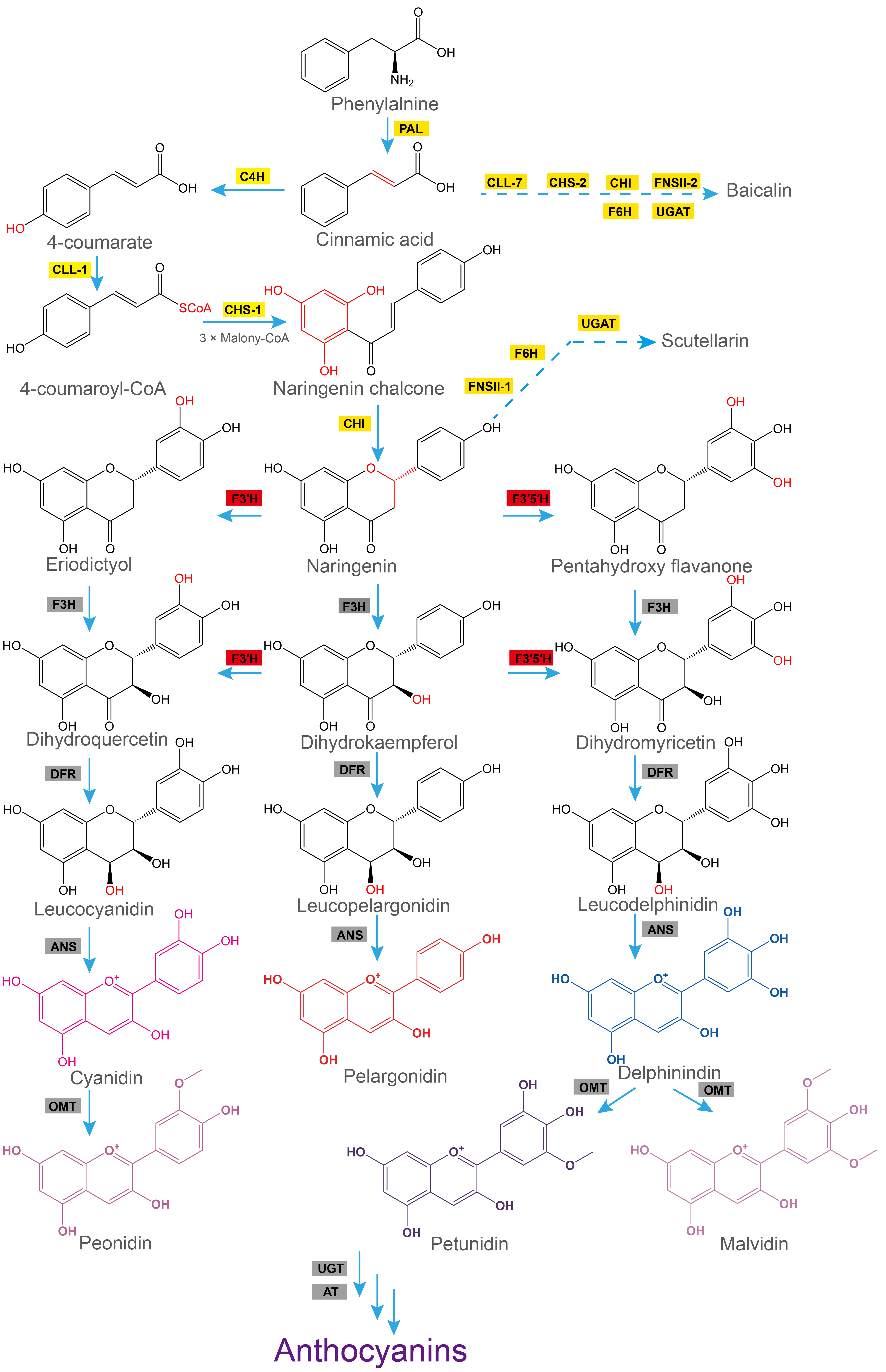
**Figure S1. The flavonoid biosynthetic pathway in *S. baicalensis***

Enzymes highlighted in yellow, red and gray indicates that their functions have been reported before, in this study or unknown, respectively. PAL, phenylalanine ammonialyase; C4H, cinnamate 4-hydroxylase; CLL-7, cinnamate-CoA ligase; CLL-1, 4-coumaryol CoA ligase; CHS, chalcone synthase; CHI, chalcone isomerase; FNSII, flavone synthase II; F6H, flavone 6-hydroxylase; F8H, flavone 8-hydroxylase; UGAT, UDP-glucuronosyltransferase; F3′H, flavonoid 3′-hydroxylase; F3′5′H, flavonoid 3′5′-hydroxylase; F3H, flavanone 3-hydroxylase; DFR, dihydroflavonol reductase; ANS, anthocyanin synthetase; OMT, *O*-methyltransferases; UGT, UDP-glucosyltransferase; AT, acyltransferase.


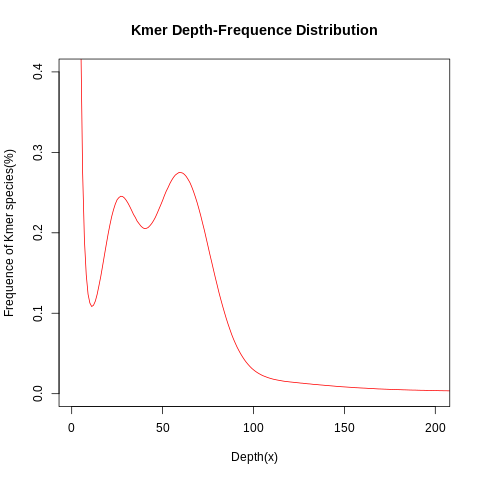


**Figure S2. Estimation of *S. baicalensis* genome size by *K*-mer analysis**

X axis shows *K*-mer depth and Y axis shows *K*-mer frequency. The genome size was measured as 356.29 Mb using this method.

**
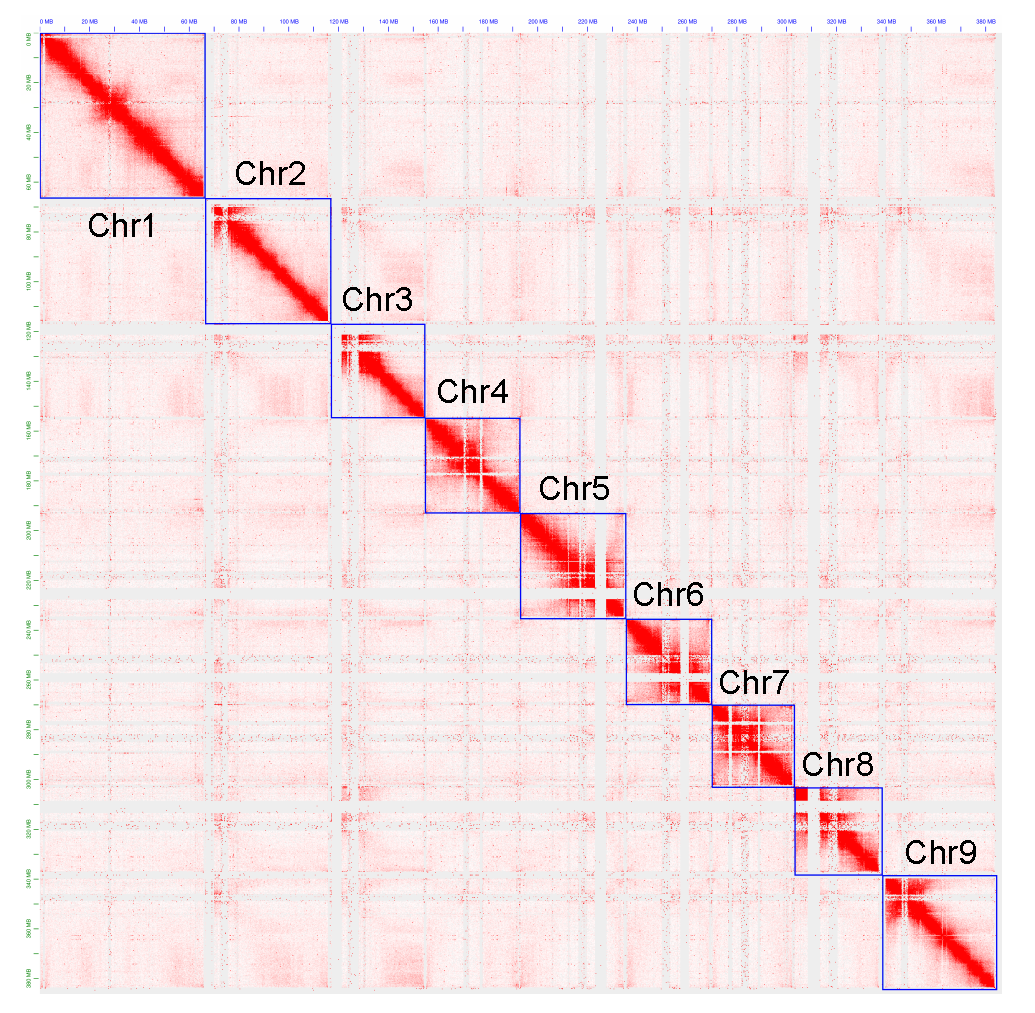
**

**Figure S3. Interaction heatmap of chromosomal fragments based on Hi-C analysis**

**
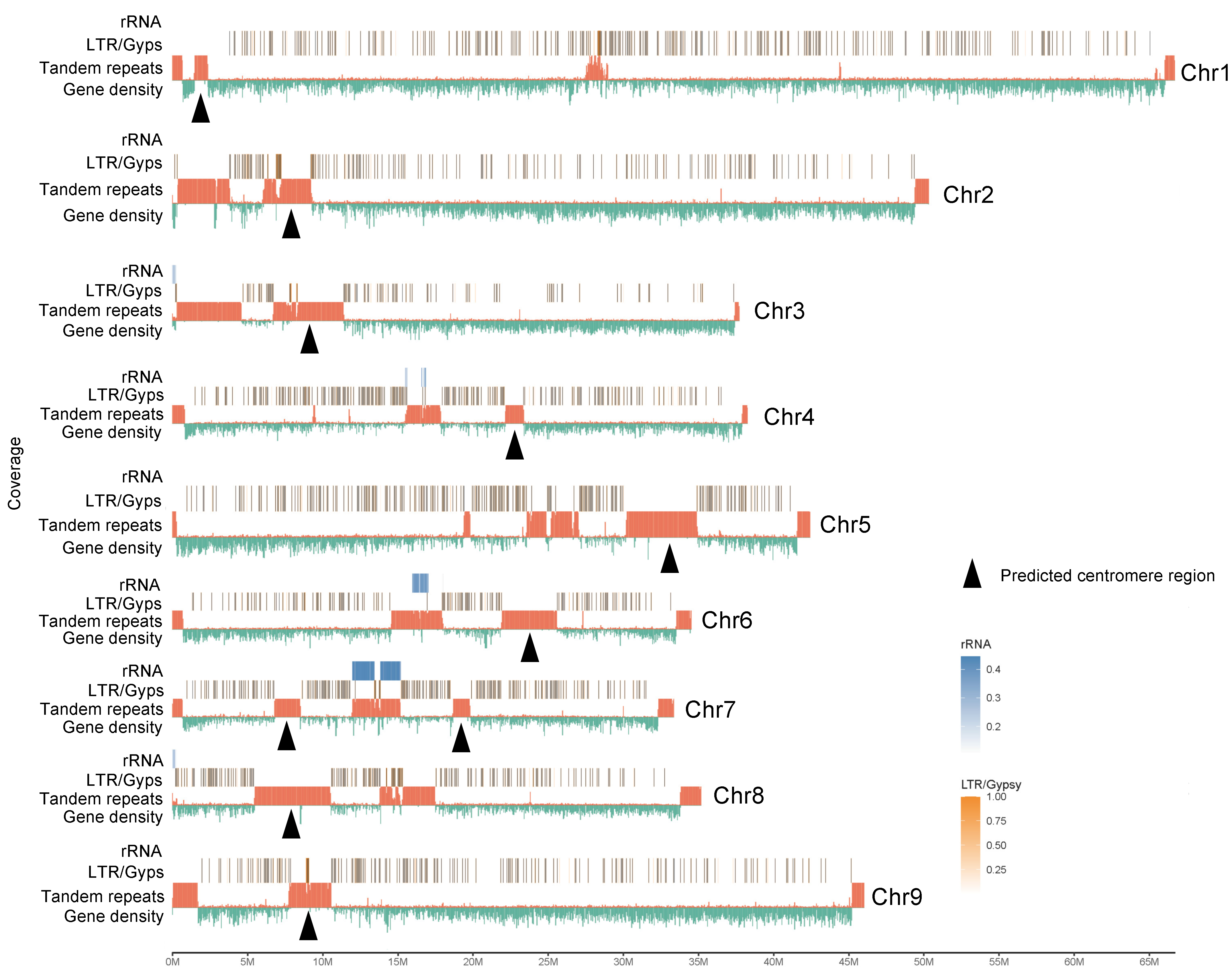
**

**Figure S4. The distribution of predicted centromere and gene density regions**

**
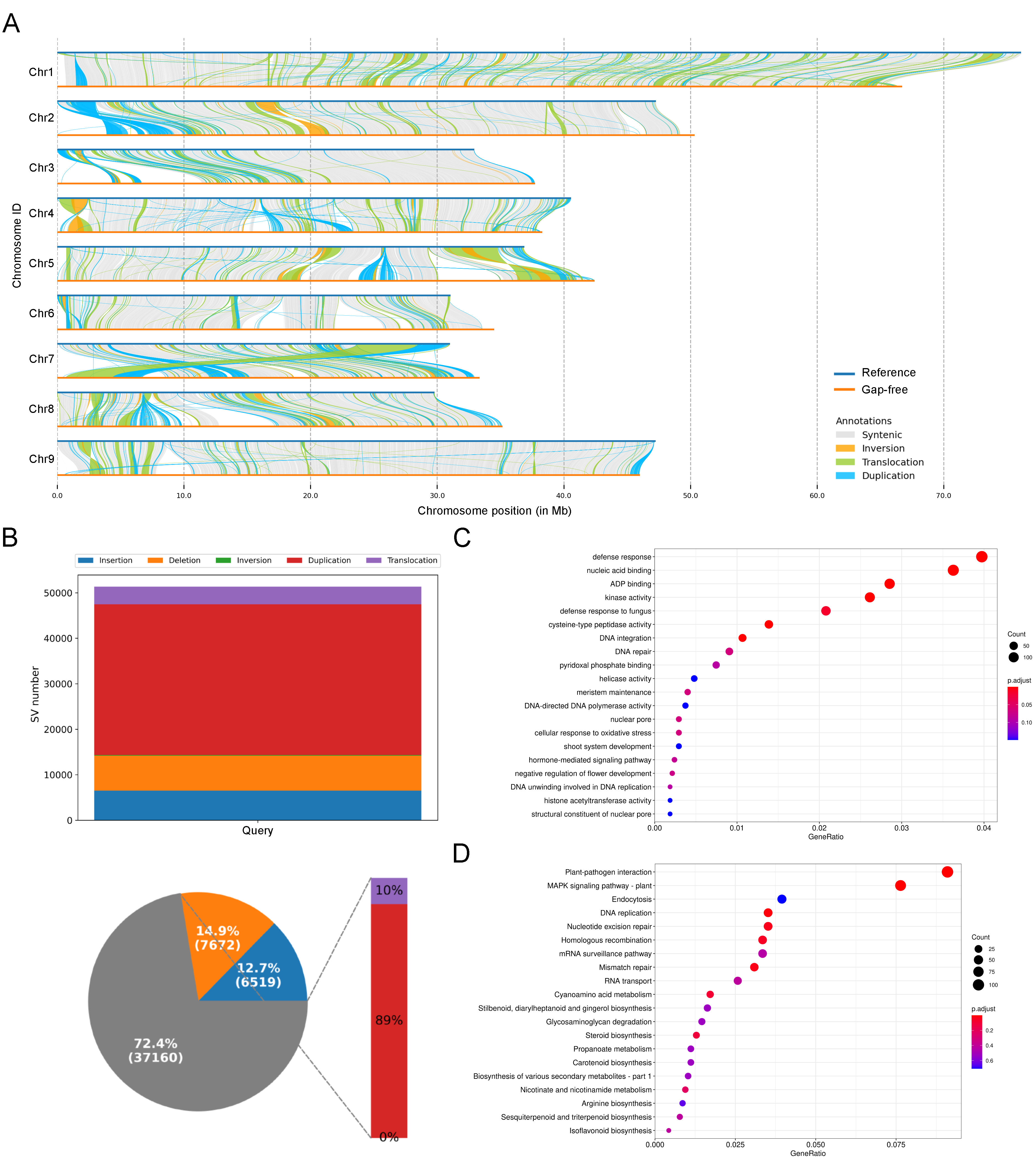
**

**Figure S5. Structural comparison between T2T gap-free genome assembly and previous genome version of *S. baicalensis***

**A.** Synteny and rearrangement identification between two genome versions. **B.** The classification and statistic of structural variations. **C.** GO enrichment of structural variations. **D.** KEGG enrichment of structural variations.

**

**

**Figure S6. GO enrichment (A) and KEGG enrichment (B) of newly added fragments in T2T gap-free genome assembly.**

**
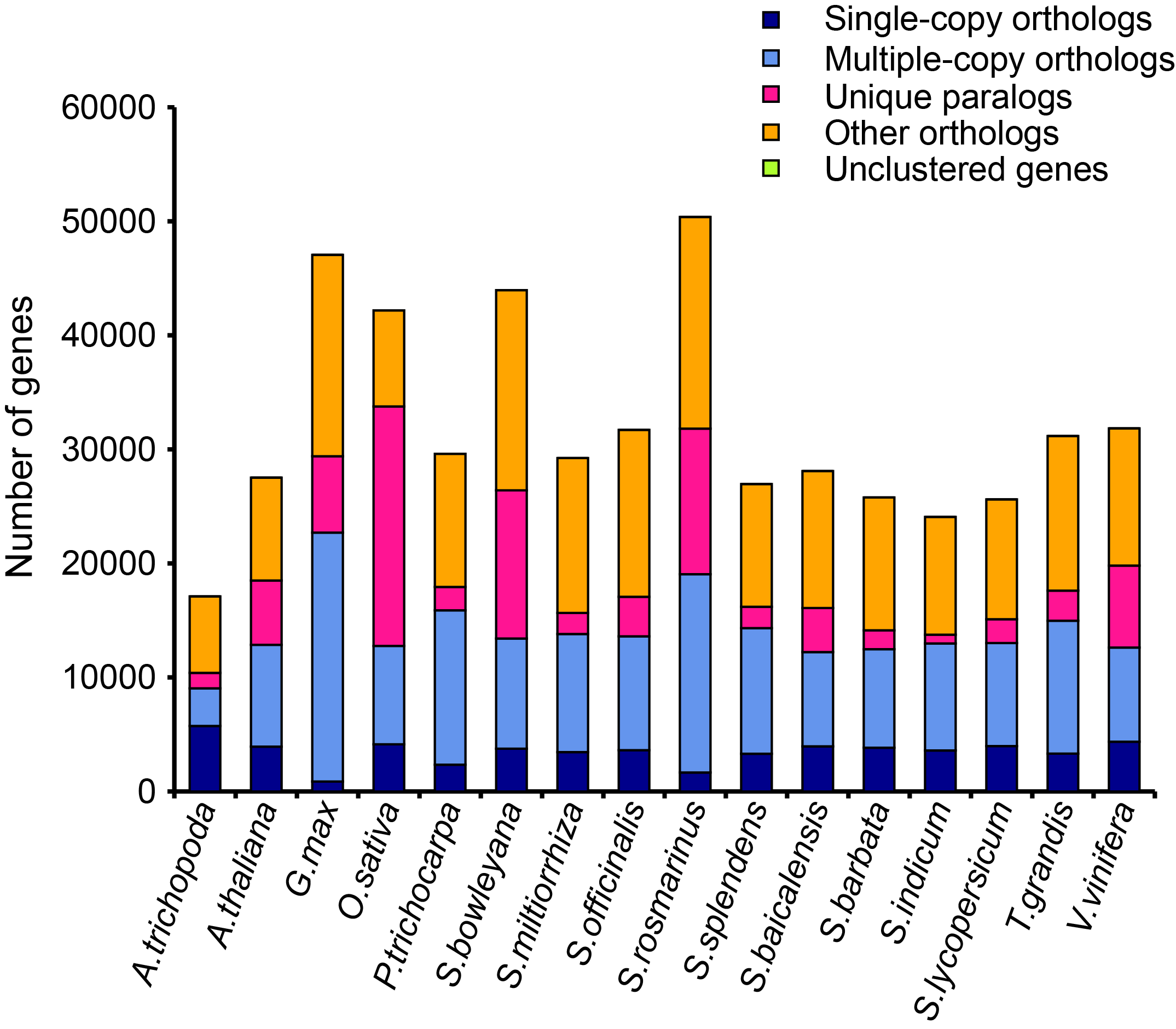
**

**Figure S7. The distribution of gene families in different species**


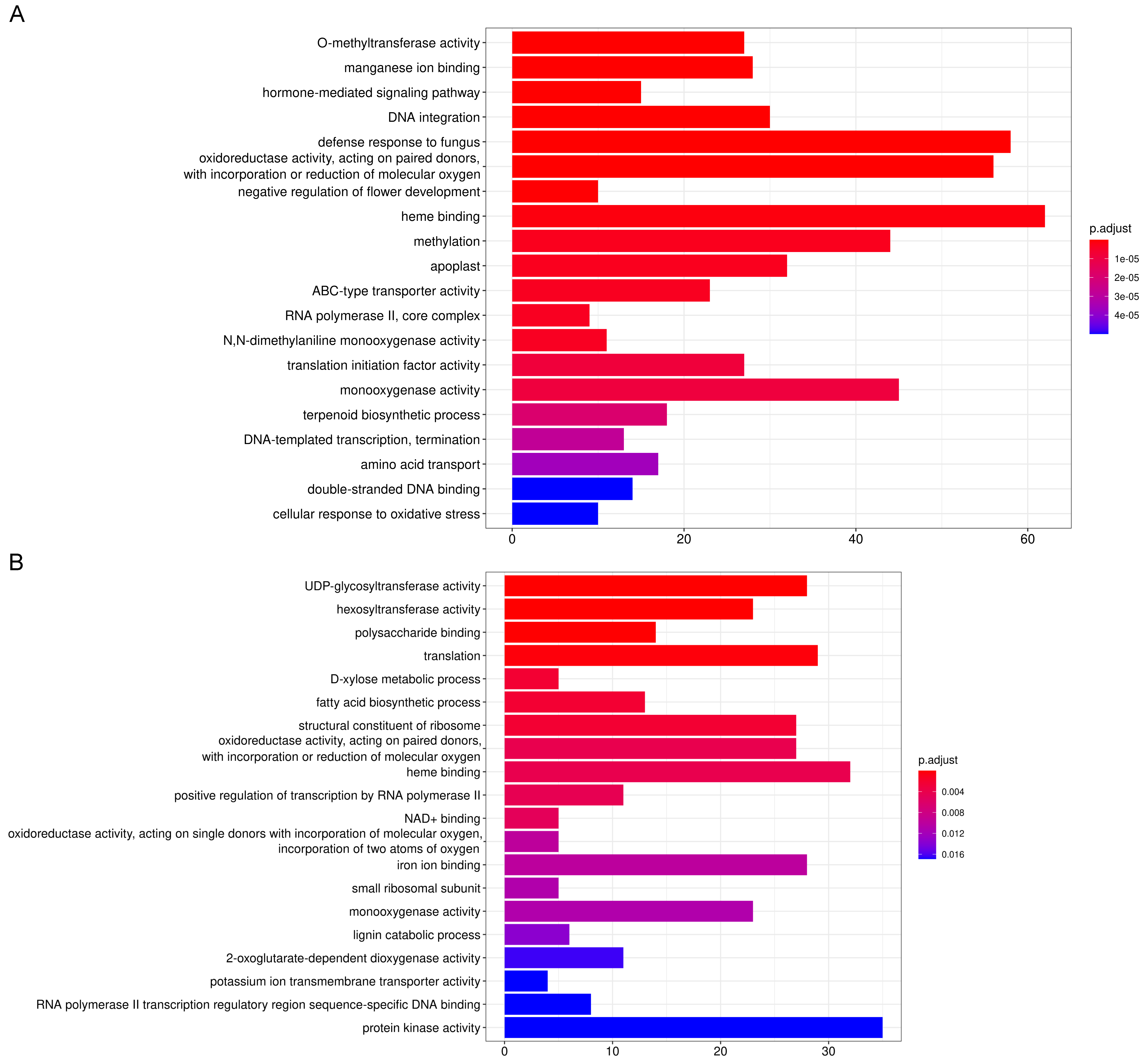


**Figure S8. GO functional enrichment of expansion and contraction genes in *S. baicalensis***

**A.** Barplot of expansion genes with GO enrichment. **B.** Barplot of contraction genes with GO enrichment.


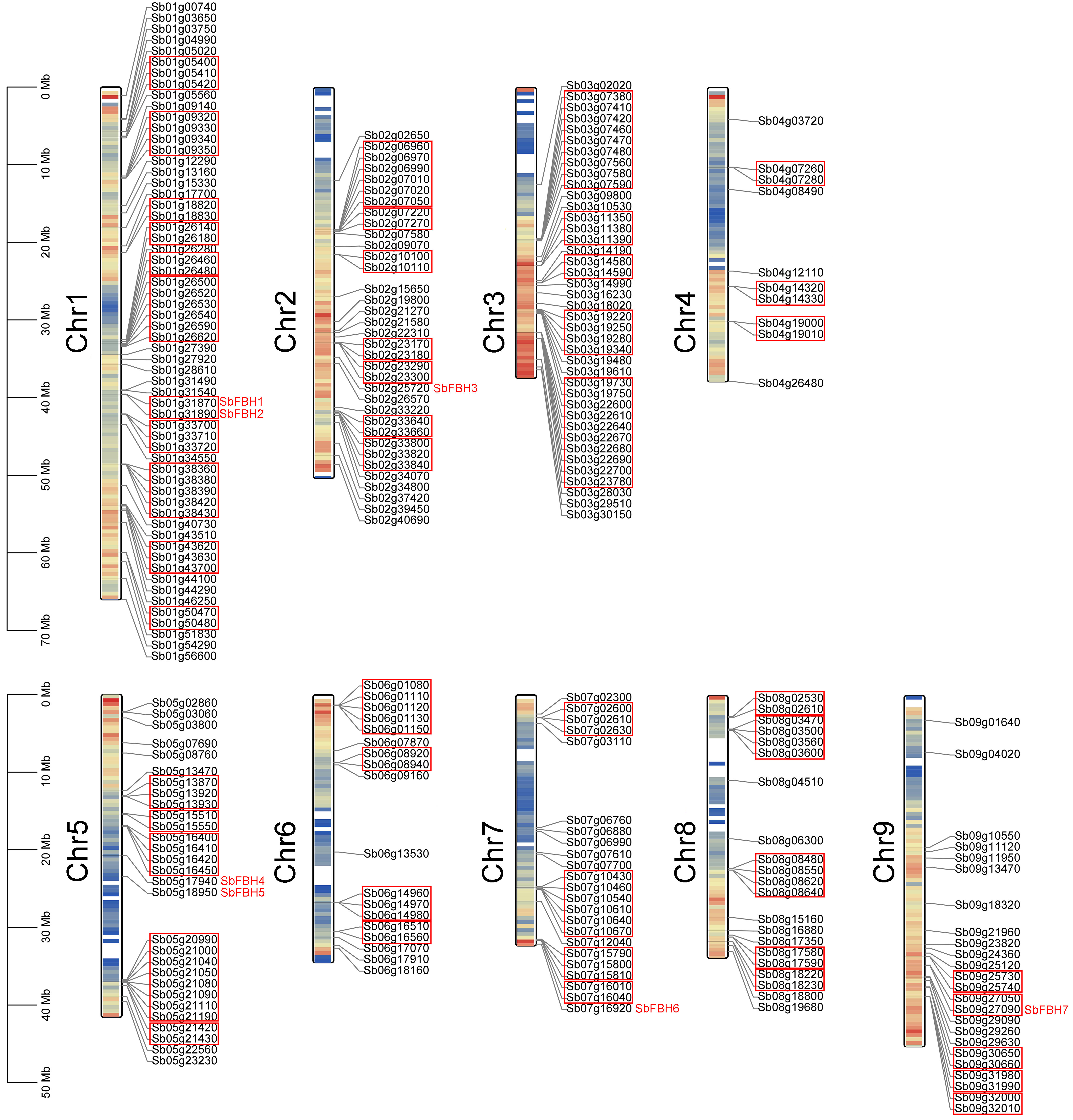


**Figure S9 Chromosomal distribution of *CYP450* genes in *S. baicalensis***

The bars show gene densities of chromosomes (Chr1-Chr9) in a 500 kb window and red rectangles represent gene clusters.


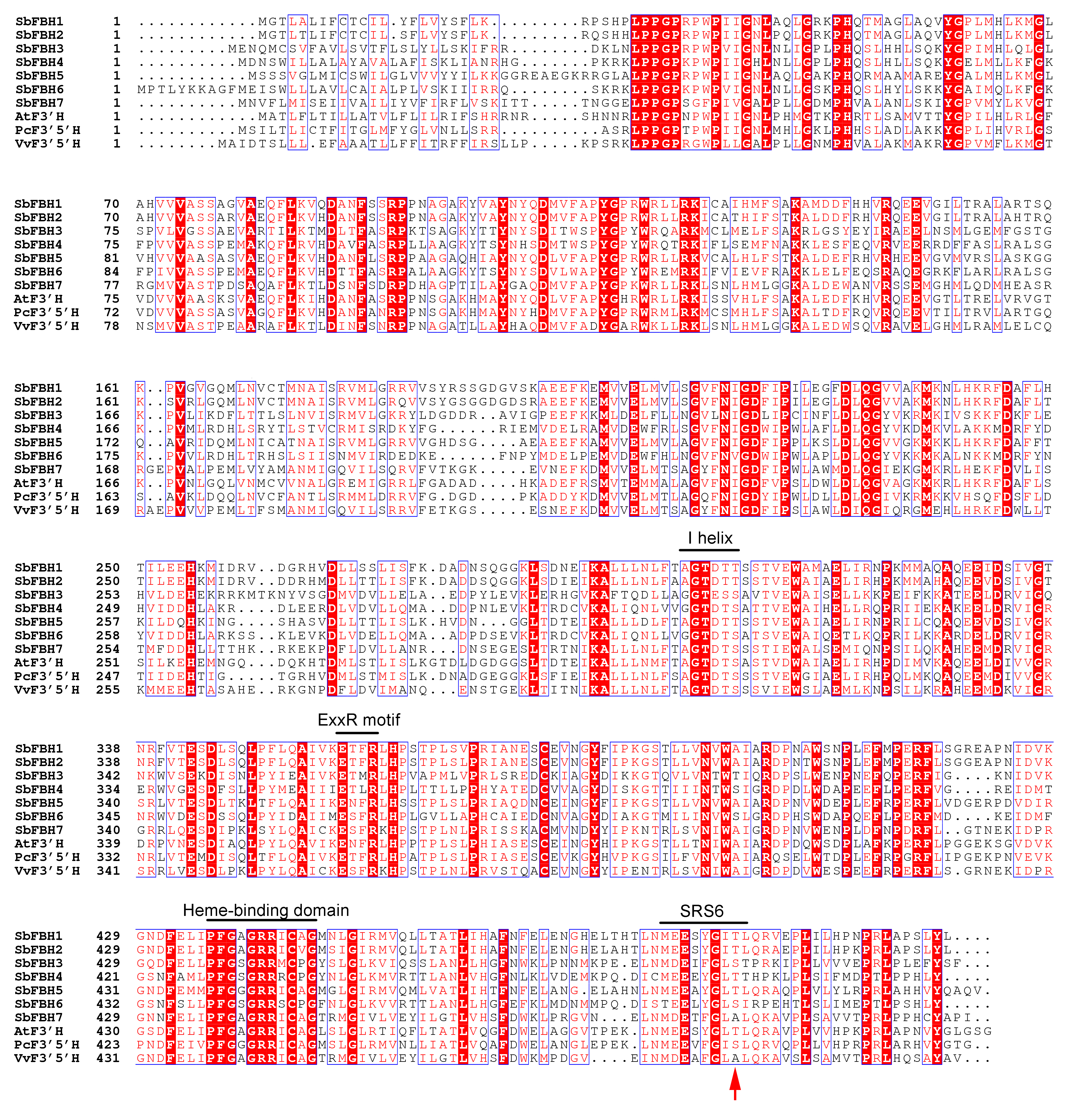
**Figure S10. Multiple sequences alignment of SbFBHs**

Amino acids highlighted in red indicated conserved site in all species, red arrow represented the key amino acid within SRS6 motif in the determination of hydroxylated function.


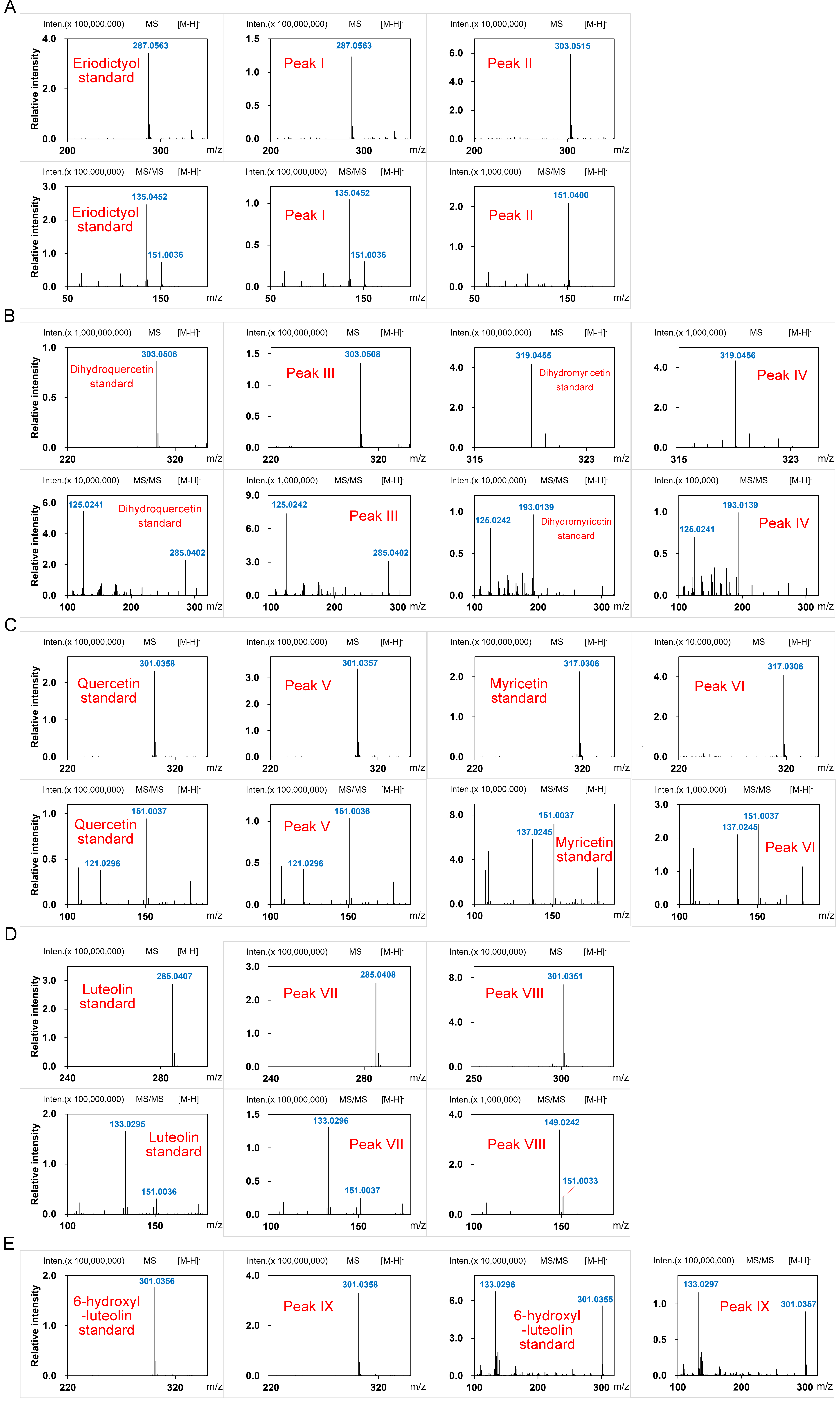


**Figure S11. MS and MS/MS patterns of standard compounds and products of yeast enzyme assays of SbFBHs**


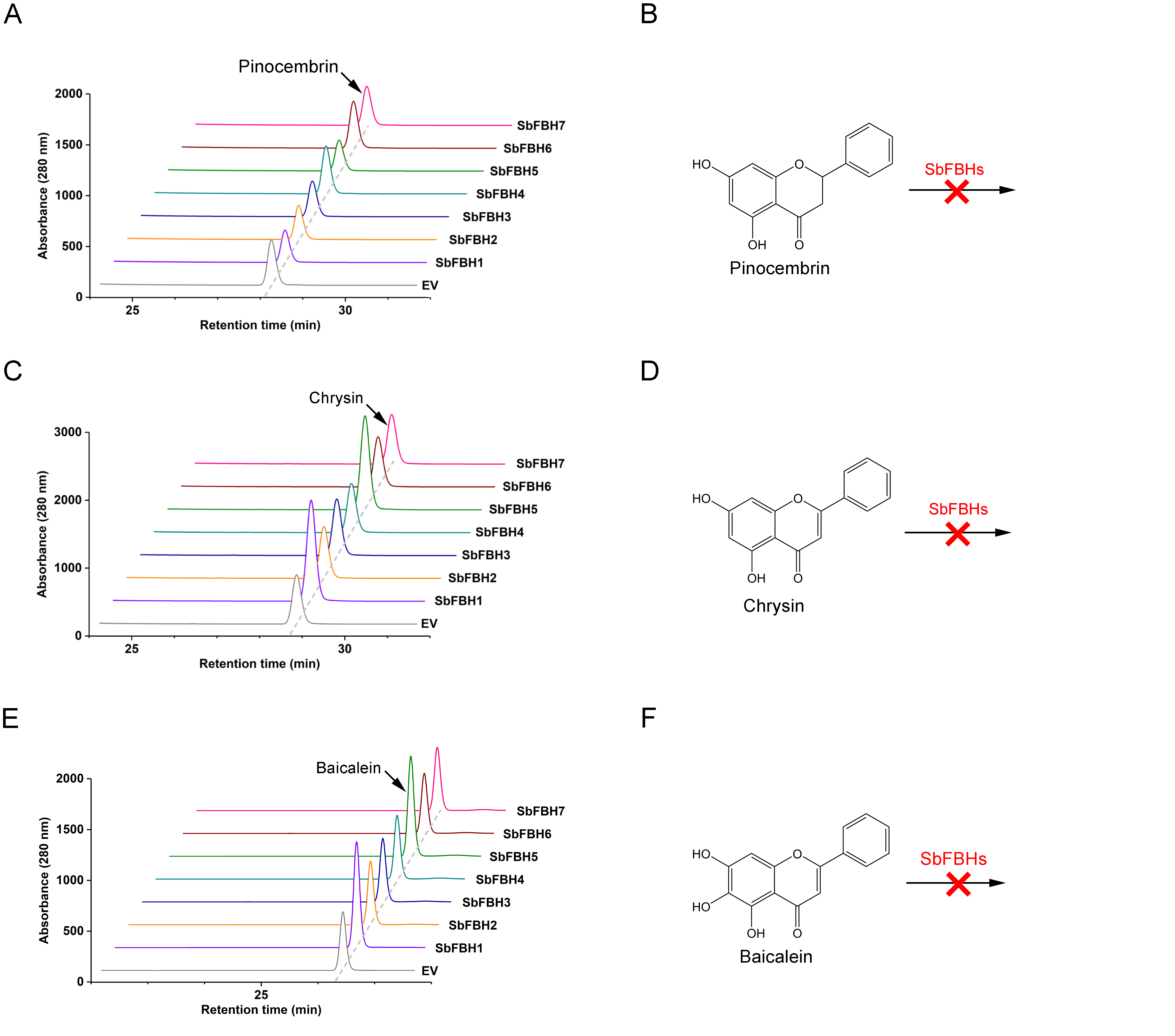


**Figure S12. Yeast enzyme assays of SbFBHs with 4′-deoxyflavonoids**

**A-F.** HPLC analysis and the reaction catalyzed by SbFBHs using pinocembrin (**A, B**), chrysin (**C, D**) and baicalein (**E, F**) as substrates in yeast enzyme assays, respectively.
